# Genome-wide association analysis revealed novel candidate genes for body measurement traits in indigenous Gudali and crossbred Simgud in Cameroon

**DOI:** 10.1186/s12864-025-11865-7

**Published:** 2025-07-14

**Authors:** Youchahou Poutougnigni Matenchi, Evren Koban Bastanlar, Matthew Hegarty

**Affiliations:** 1 Department of Animal Biosciences, Swedish University of Agricultural Sciences, Uppsala, 7023, SE-75007 Sweden; 2 Department of Biology, Ege University, Bornova, 35100 Turkey; 3 Department of Life Sciences, Aberystwyth University, Penglais Campus, Aberystwyth, SY23 3FL Ceredigion UK

**Keywords:** GWAS, Body traits, MLM, Gudali, Simgud

## Abstract

**Background:**

The genetic potential of Central African cattle for enhanced productivity remains largely unexplored. The absence of systematic pedigree recording and performance monitoring represent a major obstacle to implementing informed breeding strategies aimed at improving their production. To address this gap, we performed a genome-wide association analysis (GWAS) on a total of 856 animals genotyped with the GGP Bovine 100K array. The analysis focused on identifying genomic regions and candidate genes associated with body traits in a local Zebu (Gudali) and its crossbreed with the European Simmental (Simgud), using mixed linear models (MLM).

**Results:**

The SNP-based heritability for the four body traits studied varied between 0.23 ± 0.12 for the height at wither (HAW) to 0.44 ± 0.11 for the sacrum height (SH). The genetic correlation ranged from 0.19 ± 0.14 between height at wither and ear length (EL), to 0.81 ± 0.06 between height at wither and sacrum height. For the phenotypic correlations, the ranges were 0.58 ± 0.00 between body length (BL) and ear length to 0.90 ± 0.06 between height at wither and body length. The maximum Pairwise Linkage Disequilibrium (LD), measured as squared correlation coefficient (r^2^) was 0.465 for Gudali, decreasing by half (0.23) at a distance of 50,708 bp. For the Simgud population the maximum LD was 0.47 halving (0.23) at 99,201 bp. Notably, we observed extended LD patterns across both the Gudali and Simgud genomes, persisting over distances greater than 1 mbp. These features hold significant potential for association analysis studies and genetic improvement initiatives. A total of 52 SNPs were identified has being associated to the considered body traits. These SNPs were mapped within or near 70 candidate genes across the genome. Among them, the *ADGRD1*, *NDUFAF1*, *RTF1* and *ITPKA* genes exhibited a pleiotropic effect as they were associated with two or more traits. Additionally, *LAMTOR5, PCDH9, BCL2, CTIF, BHLHA15, UNC5D, CNTNAP5, TMEM109, TMEM132A*, and *NOS1AP* genes showed direct association with individual body traits.

**Conclusions:**

This study identified a number of novel loci associated with pathways influencing growth and body traits, disease resistance and immunity, reproduction and milk production. Overall, the identified genes could be considered as candidate genes in any attempt to improve growth, disease resistance and production in tropical cattle raised under extensive management systems. These genes or genomic regions should be prioritized in future cattle breeding programs in Cameroon.

**Supplementary Information:**

The online version contains supplementary material available at 10.1186/s12864-025-11865-7.

## Introduction

Morphometric traits are routinely used as performance indicators for selection initiatives aiming to improve beef cattle production [1]. They not only exhibit moderate to high heritability [2, 3], but also are highly correlated [4–6] with major traits of economic importance such as reproduction [7], longevity [8–11], carcass traits [2], body weight [12], growth [13], animal welfare [4] and health [14]. Body trait measurements hold great promise for animal improvement, especially where routine pedigree and performance record keeping is lacking, as seen in most African breeding systems. As with most traits of economic importance in farm animals, body measurements are controlled by many genes with small contribution and also influenced by environmental conditions [15, 16]. Traditional methods of selection would lead to limited improvement in these traits [17]. Genomic technologies offer good opportunities for breeding programs in African countries where the local adapted breeds are not well characterized for their performance traits [18]. They can be valuable in this case in assessing breed composition and parentage assignment [19, 20]. Moreover, genomic technologies can help identify highly performing, disease resistant animals that could be subjected to precision breeding to produce and disseminate improved elite offspring. It is now possible to use genome-wide scanning tools to characterize cattle populations [21], perform studies of association [22] and detect signatures of selection for productivity [23] as well as genomic evaluation [24].

Among genomic technologies, genome-wide SNP arrays are a powerful tool for identifying associations between genetic variants and phenotypic traits (GWAS), as well as for analyzing breed composition and genomic structure in animals. These technologies are routinely used in America, Europe, and Asia. GWAS was first developed and applied to human disease research and has since driven major breakthroughs [25]. The principle makes use of sequence variants (mainly single-nucleotide polymorphisms, i.e. SNPs) across the entire genome, along with phenotype and lineage information, to perform association analysis and identify genes or regulatory elements important for targeted traits. Compared to traditional QTL mapping strategies, GWAS provides major advantages, mainly in its power to identify narrowed genomic regions harboring causal variants [26]. GWAS could therefore be considered as an ideal technique for discovering genes underlying complex traits and offers significant benefits for countries aiming to develop sustainable agriculture strategies and increase yields. However, these studies, especially in cattle, are still limited in their use in the African continent, partly due to lack of technology, lack of trained personnel, limited resources, poor infrastructure, difficulties with phenotypic data, lack of record keeping and crossbreeding of animals. The fast decrease in genotyping and sequencing costs opens an avenue for routine evaluation of breeds in Africa using genome-wide analysis. Several recent studies have been conducted in Africa using genomic tools for genome-wide characterization, parentage assignment [27], and breed composition [28]. These surveys have been generally conducted in West Africa [29] and East Africa [30]. One such study identified several candidate genes associated with body traits such as PIK3R6 and PIK3R1 in four cattle breeds of Benin [31].

In Cameroon, the benefits of genomic technology are not yet perceptible, and research has been limited to characterization using microsatellites [32]. The only genome-wide analysis of local cattle of Cameroon was conducted on a single sample per breed [33]. To the extent of our knowledge, no genome-wide association study has yet been conducted in cattle from Cameroon. Among the local breeds of Cameroon, Gudali Zebu is the most popular local breed, especially among small farmers in the Adamawa plateau [34]. Also known as Peulh or Fulbe zebu, Gudali - because of its well-known meat and milk production potential [35] - has always been at the centre of cattle improvement initiatives in Cameroon. Similar in conformation, size and origin to the East African shorthorned zebu, it is a well-tempered animal endowed with good adaptation to poor management and harsh environments. It produces quite well under low input systems [36] and thrives under Cameroon’s disease-loaded agroecological conditions. Improvement schemes have aimed to combine this local adaptivity with the higher production of European taurine cattle. Gudali cattle were used in the development of the wakwa hybrid through crossbreeding with American Brahmans [37] and more recently in the creation of the Simgud -a cross between the Italian Simmental and Gudali- in the ranches on the National Livestock Company (SODEPA).

To investigate the genomic background of productivity traits in the Gudali and the crossbred Simgud, we present here the results of a GWAS study of 856 animals (717 Gudali, 139 Simgud), along with analysis of population structure and linkage disequilibrium. We show significant marker-trait associations with four body measurements highly correlated with animal productivity, representing a resource for genomic improvement efforts in Cameroon cattle breeding.

## Results

### Phenotypic description

The results show breed differences in morphological traits between Gudali and Simgud, and also substantial variation within the two breeds. The descriptive statistics for body measurements in Gudali and Simgud are presented in Table 1. The mean values of all the traits were 104.61 cm, 134.00 cm, 130.98 cm and 21.82 cm for body length, height at wither, sacrum height and ear length respectively. Likewise, the coefficient of variation ranged between 13, 7, 8 and 12 respectively for body length, height at wither, sacrum height and ear length. The distribution of the four traits and the multifactor ANOVA analysis (Additional file 7), present the various factors influencing the traits considered. The height at wither was significantly influenced by ranch, camp, herd (*p* < 0.001) and sex (*p* < 0.1). The factors ranch, camp and herd significantly affected (*p* < 0.001) sacrum height, as did the sex and age factors at (*p* < 0.01) and (*p* < 0.1) respectively. Ear length was significantly (*p* < 0.001) affected by ranch and herd.

**Table 1:** Descriptive statistics for the phenotypic traits considered

Trait	Mean	SD	Min	Max	CV%
BL	104.61	13.20	80.00	153.00	13
HAW	134.00	9.41	93.00	170.00	7
SH	130.98	10.13	86.00	164.00	8
EL	21.82	2.60	15.00	30.00	12

Note: *BL* Body length, *HAW* Height at wither, *SH* sacrum height, *EL* ear length

### Phenotypic, genetic correlation and heritability estimates

The four measured body traits displayed strong phenotypic and genetic correlations with one another, and exhibited moderate to high heritability. The genetic, phenotypic correlations as well as the heritability results are shown in Table 2. The heritabilities were moderate, ranging from 0.23 ± 012 for the height at wither to 0.44 ± 0.11 for the sacrum height. The results show that the four body traits under study are strongly correlated. The phenotypic correlations were moderate to high, ranging from 0.58 ± 0.00 between body length and ear length to 0.90 ± 0.06 between body length and height at wither. The same tendency was generally observed for the genetic correlations, which ranged from 0.19 ± 0.14 between ear length and body length, to 0.81 ± 0.06 between height at wither and sacrum height.

**Table 2:** Genetic and phenotypic correlations and heritability estimates for the traits considered

Trait	BL	HAW	SH	EL
**BL**	**0.27 ± 0.10**	0.49 ± 0.12	0.43 ± 0.13	0.19 ± 0.14
**HAW**	0.90 ± 0.06	**0.23 ± 0.12**	0.81 ± 0.06	0.72 ± 0.09
**SH**	0.74 ± 0.00	0.88 ± 0.00	**0.44 ± 0.11**	0.65 ± 0.10
**EL**	0.58 ± 0.00	0.71 ± 0.001	0.72 ± 0.00	**0.24 ± 0.10**

Phenotypic correlation (below diagonal), genetic correlation (above diagonal) and heritability (in bold) between traits

### Population genetic analysis

**Fig. 1:**
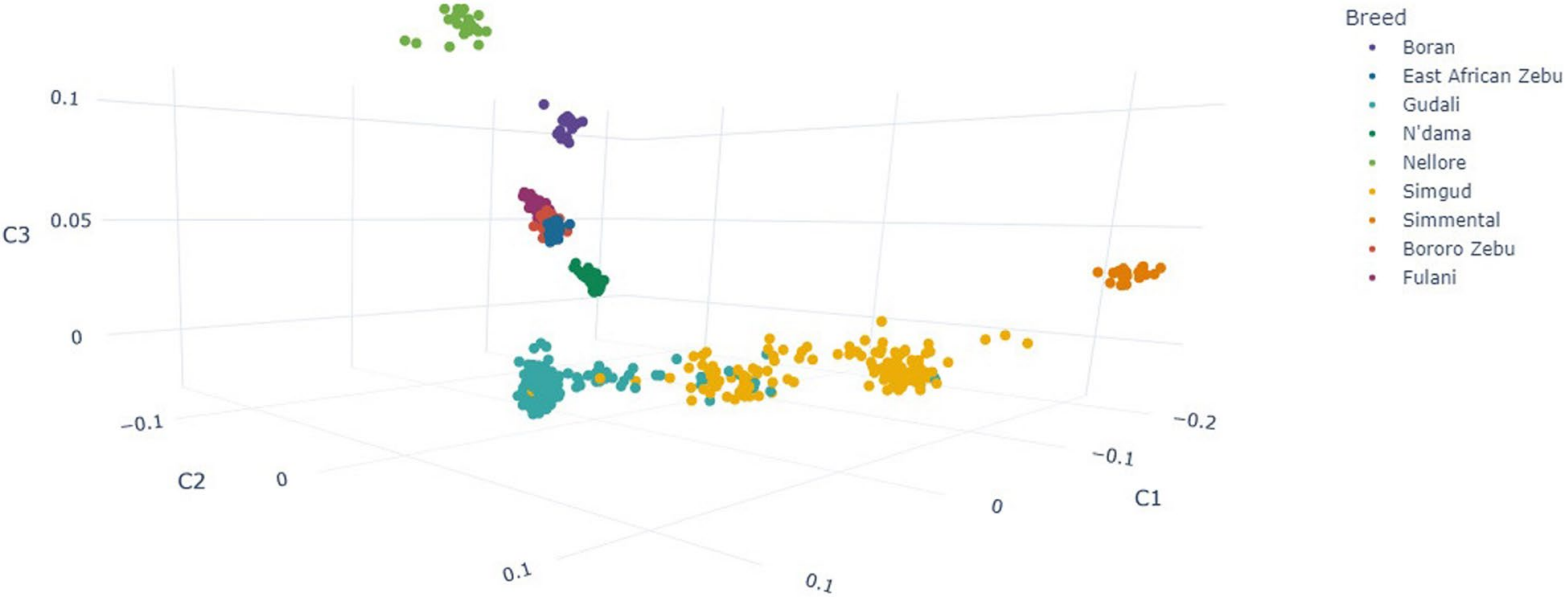
Multidimensional scaling (MDS) plot of relationships between Gudali, Simgud and reference populations from the WIDDE database

**Fig. 2:**
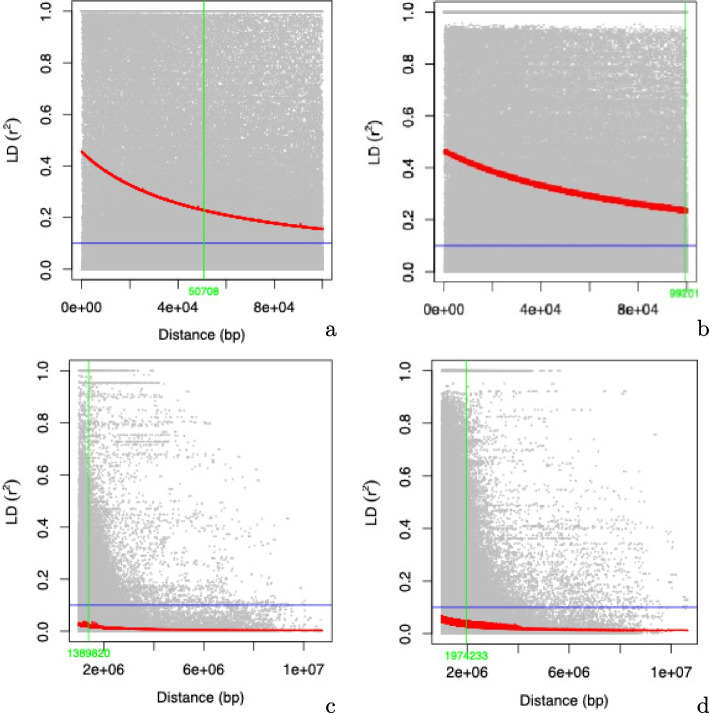
The linkage disequilibrium (LD) decay analysis at distances below 100 kbp in Gudali (**a**), Simgud (**b**) and at distances above 1 mbp in Gudali (**c**) and Simgud (**d**)

There is a clear distinction between Gudali and Simgud populations. Figure 1 presents the multidimensional scaling (MDS) plot of relationships between Gudali, Simgud and the reference populations. The Simgud population appears to cluster into two subsets of about 25% and 50% between the reference Simmental and the local Gudali. The maximum pairwise linkage disequilibrium estimates show a general decline with the marker distance but persisted up to distances over 1 mbp. Figure 2 illustrates the evolution of LD throughout the Gudali and Simgud genomes. The maximum LD is 0.465 in Gudali breed and decreased by half (0.23) at a distance of 50,708 bp, while for the Simgud population the maximum LD is 0.47 and decreased to half at 99,201 bp. Sliding across the genome to appreciate the evolution of LD, a general decrease with the marker distance was observed. Interestingly, we observed long ranges of LD across the genome spanning distances of over 1 mbp distance. These features hold great potential interest in association analysis.

### Genome-wide association analysis

**Fig. 3:**
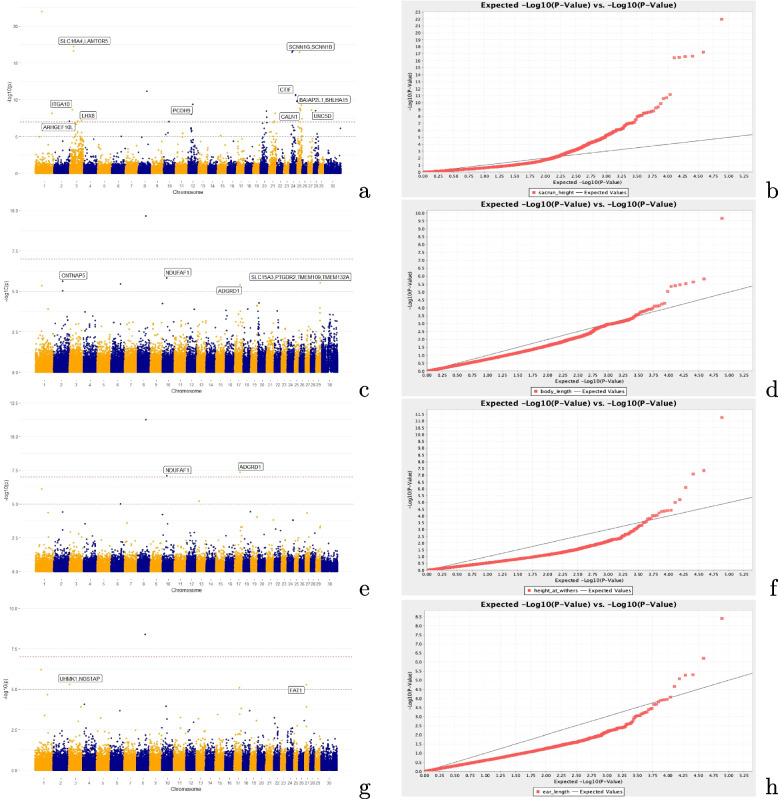
Manhattan and QQ plots of the Sacrum Height (**a**, **b**), Body length (**c**, **d**), Height At Wither (**e**, **f**) and Ear Length (**g**, **h**). The horizontal coordinates represent the chromosomes, and the vertical coordinates are -log_10_ (P) values for each marker

The GWAS was performed using the MLM approach to account for relationship between individuals as well as population structure. Figure 3 presents Manhattan and QQ plots of the association between SNPs and the four traits considered. The genome-wide significant SNPs found associated to body traits in Gudali and Simgud cattle are shown in Table 3. Overall, a total of 52 SNPs were found significantly associated with body traits. Among them, 33 were related to sacrum height and located in or near 51 genes, 8 were linked to body length and mapped in or near 14 genes. For ear length, we identified 5 SNPs and mapped them in genomic regions harbouring 6 genes, and another 6 SNPs identified were associated to height at wither and mapped in or near 7 genes. Some SNPs showed significant associations with more than one trait, suggesting pleiotropic effects. Among these, the SNP BovineHD1700013218 on BTA17:46,179,818 bp mapped within *ADGRD1* gene showed significant association with body length, height at wither and ear length. On BTA10:36,829,871 bp, the SNP ARS-BFGL-NGS-531 was significantly associated with body length ($p < 10$^−6^) and height at wither ($p < 10$^−8^). The location of this SNP falls whithin the gene *NDUFAF1*, near five other genes including *CHP1, OIP5, NUSAP1, RTF1, ITPKA*. For sacrum height-specific associations, the most significant SNPs detected included seven on BTA25, six on BTA3 and five on BTA24. On BTA3, the SNPs BovineHD0300010326 and BovineHD0300010324 were located in close proximity (2.4 kbp) to each other and exhibit the most significant association ($p = 6.08E$^−18^) and ($p = 2.37E$^−17^) with the trait respectively. This genomic region harboured five genes including *KCNA10, CYM, PROK1, LAMTOR5, SLC16A4* and *RBM15*. The most significant locus observed on BTA24 was assigned to *CCDC178* and *CELF4*. Similarly, the only strongly associated ($p = 3.69E$^−17^) locus on BTA25 was mapped to SCNN1B, located approximately 90 kbp downstream of SCNN1G. On the same chromosome, the SNPs BovineHD2500008133 at position 28,988,722 bp and ARS-BFGL-NGS-101637 at 28,648,581 bp are both located within the *CALN1* gene and strongly associated ($p = 3.01E$^−9^) and ($p = 9.91E$^−8^) respectively with sacrum height. For body length, the two most significant SNPs (Hapmap52707-rs29020755 and BTB-00103137) observed on BTA2 were located within the *CNTNAP5* gene. Another significant locus (ARS-BFGL-NGS-15883) was found on BTA29 and mapped to *SLC14A3*, near *PTGDR2, TMEM109* and *TMEM132A* genes. A total of 6 SNPs were significantly associated with height at wither. among them, the SNP ARS-BFGL-NGS-531 and BovineHD1700013218 were already mentioned for their association with more than one trait under study. For the ear length, the most significantly ($p = 4.98E$^−6^) associated SNP BTA-69126-no-rs is located within a genomic region harbouring four genes including *NOS1AP* and *UHMK1*. On BTA27:16,529,725 bp, the most significant locus was assigned to the *FAT1* gene. Following imputation to the higher-density African reference dataset, a total of 140 SNPs were found associated with sacrum height (86 SNPs), body length (14 SNPs), height at wither (25 SNPs) and ear length (15 SNPs). The identified SNPs and candidate genes are listed (Supplementary Table A1) and the corresponding Manhattan plots are presented in Supplementary (Fig. S4).

**Table 3:** Genome-wide significant SNPs and associated candidate genes

Trait	SNP	CHR	BP	*P*-value	Within gene	<100kb
**Pleiotropic genes**						
BL	BovineHD0100015405	1	54281520	4.46E-06		
EL				6.13E-07		
HAW				7.80E-07		
SH				1.03E-22		
BL	BovineHD0600022959	6	81745649	3.57E-06		
HAW				9.87E-06		
BL	BTA-81825-no-rs	8	73015373	2.21E-10		
EL				4.05E-09		
HAW				5.44E-12		
SH				6.93E-12		
BL	ARS-BFGL-NGS-531	10	36829871	1.50E-06	NDUFAF1	CHP1, OIP5, NUSAP1, RTF1, ITPKA
HAW				8.07E-08		
BL	BovineHD1700013218	17	46179818	3.88E-06	**ADGRD1**	
EL				8.12E-06		-
HAW				4.51E-08		
**Body length**						
	Hapmap52707-rs29020755	2	76976290	2.32E-06	CNTNAP5	
	BTB-00103137	2	76839374	9.35E-06	CNTNAP5	
	ARS-BFGL-NGS-15883	29	37283656	2.88E-06	SLC15A3	PTGDR2, PRPF19, TMEM109, TMEM132A, CD6
**Ear length**						
	BTA-69126-no-rs	3	7057469	4.98E-06		UHMK1, SH2D1B, NOS1AP, SPATA46
	chr27_15586715	27	16529725	5.19E-06	FAT1	
**Height at withers**						
	BovineHD1300006489	13	21894567	6.28E-06		
**Sacrum height**						
	BovineHD0100042173	1	144595788	7.22E-09		KRTAP10-8, KRTAP12-2, UBE2G2, SUMO3
	BovineHD0200039720	2	135082967	8.32E-08	ARHGEF10L	
	BovineHD030010326	3	33009094	6.08E-18		
	BovineHD0300010324	3	33006680	2.37E-17		PROK1, LAMTOR5, KCNA10, CYM, RBM15
	BovineHD0300006766	3	21474502	2.26E-09	ITGA10	TXNIP, POLR3GL, ANKRD34A, RBM8A, GNRHR2, PEX11B, ANKRD35, PIAS3, NUDT17, POLR3C, RNF115
	ARS-BFGL-NGS-2973	3	101700489	6.63E-08	ERI3	bta-mir-2414
	BovineHD0300020629	3	69700962	7.42E-08		LHX8
	Hapmap51849-BTA-68314	3	69713184	8.99E-08		
	ARS-BFGL-NGS-97032	10	44727501	8.98E-08	GNG2	
	BovineHD1200011574	12	40470693	4.22E-10	PCDH9	
	BovineHD12000008682	12	29404264	8.24E-09		
	BovineHD1200008688		29441999	8.87E-09		
	Hapmap24428-BTA-112183	20	53024269	3.54E-09		
	BTB-01128234	20	54554943	2.42E-08		
	Hapmap50255-BTA-119714	21	54304967	7.09E-09		
	BTB-01452384	21	50156924	8.79E-08		
	BovineHD2400006667	24	24259870	2.43E-17		CCDC178
	ARS-BFGL-NGS-117961	24	19726696	3.50E-17	CELF4	
	BovineHD2400013697	24	48525522	2.04E-11	CTIF	
	ARS-BFGL-NGS-118412		48528658	2.59E-11		U6, SMAD7
	ARS-BFGL-NGS-73573	24	61367470	1.45E-10		PHLPP1, BCL2
	ARS-BFGL-NGS-14982	24	30132541	9.56E-09		
	ARS-BFGL-NGS-7030	25	20942828	3.69E-17		SCNN1G, SCNN1B
	BovineHD2500010674	25	37709326	5.68E-10	BAIAP2L1	BRI3, TECPR1, BHLHA15, LMTK2
	BovineHD2500006500	25	22931035	1.54E-09		ZKSCAN2
	ARS-BFGL-NGS-78220	25	28385587	1.93E-09	TMEM248	SBDS
	BovineHD2500008133	25	28988722	3.01E-09	CALN1	
	ARS-BFGL-NGS-101637		28648581	9.91E-08		
	ARS-BFGL-NGS-18654	25	41015047	3.73E-08	MAD1L1	SNX8, NUDT1, MRM2
	ARS-BFGL-NGS-107550	27	31299640	2.42E-09	UNC5D	
	Hapmap43636-BTA-63692	28	18703012	3.19E-09		

### Enrichment analysis

The enrichment analyses performed have improved our understanding of the functions of identified candidate genes. Two enriched regions (Supplementary Fig. S5) were found on BTA3 and BTA25. The KEGG enrichment analysis has revealed that the candidate genes identified in our study mainly participate in the aldosterone-regulated sodium reabsorption and taste transduction pathways (Supplementary Fig. S6), as well as ubiquitin mediated proteolysis and protein sumoylation. Candidate genes *SUMO3, PIAS3, SCNN1G, SCNN1B, PRPF19, UBE2G2* were found to be involved in these pathways, indicating their role in controlling body size through regulation of various metabolic and biological processes.

## Methods

### Sampling

Samples were collected from various herds across three agroecological zones of Cameroon (Supplementary Fig. S1). In the Faro ranch sampling was carried out across four camps (Bangone, Male I, GMB, Male 8) with animals selected randomly from 16 different herds. In the Ndokayo ranch, samples were collected from three camps (Songongo, Camp general, Minale) covering a total of nine herds. At the Jakiri ranch, sampling was limited to one camp with animals drawn randomly from six herds (AI1, AI2, AI3, Bull herd, AI5, Heifer). The animals were categorized into three age groups: young (1-4 years), adults (4-8 years) and old (over 8 years). Representative images of the Gudali and Simgud are shown in Supplementary Figs. S2 and S3 respectively. A total of 856 animals were sampled and blood samples collected from the jugular vein into EDTA tubes using sterile 5 ml syringes. In the field, samples were stored at −4^o^C and later transferred to –20^o^C for long-term storage. DNA extraction was subsequently carried out at EGE University (Turkiye).

### Linear body measurements

Morphometric traits were measured on Gudali and Simgud while they stood on a flat surface, using a standard tailor’s measuring tape and a measuring stick. The recorded measurements included body length (BL), height at wither (HAW), sacrum height (SH) and ear length (EL). These traits were selected based on their potential and demonstrated association with key production traits as well as their proven correlation with growth performance and disease resistance [8, 9].

### Genotyping and quality control

DNA was extracted using a chloroform-based protocol adapted from Guha et al., (2018) [38]. The quality and quantity of the extracted DNA were assessed using a NanoDrop™ 2000 spectrophotometer and confirmed by 0.8% TBE agarose gel electrophoresis. A total of 856 DNA samples with optimal concentration and purity were genotyped using the GeneSeek Genomic Profiler (GGP) Bovine 100 K assay (http://www.neogen.com/geneseek/). Quality control of the resulting dataset was performed using PLINK software v.1.07 [39] with filtering thresholds set as follows: minor allele frequency (MAF) < 0.05; maximum SNP missingness < 0.1 and maximum individual missingness < 0.1. A call rate of 0.99 was achieved. Of the 95,256 SNPs included in the GGP 100 K array, 77,242 SNPs passed the filter and were retained for subsequent analysis.

### Control of environmental and genetic structure effects in the population

The body traits considered were first tested for normality followed by one-way analysis of variance (ANOVA) to assess trait-wise significance. Subsequently, a multifactor ANOVA was conducted in R (R Core Team, 2023), incorporating the factors: breed, ranch, camp, herd, age, and sex. For population structure assessment, the filtered SNP dataset was merged with additional reference populations from the WIDDE database [40], including 24 Nellore, 20 Simmental, 20 Boran, 20 East African Short-horn Zebu, 56 N’dama, 23 Bororo Zebu and 43 Fulani individuals. An identity-by-state (IBS) genomic relationship matrix was generated using the stratification option in PLINK. This matrix was used to perform a multidimensional scaling (MDS) analysis in PLINK, and the first 20 MDS components (Additional file 8) were fitted in the model as covariates in the GWAS to correct for population stratification.

### Estimation of phenotypic, genetic correlations and heritability

A descriptive analysis of the four traits considered was performed using R software. The key statistics calculated included the minimum, maximum, mean, standard deviation, and coefficient of variation. In addition, phenotypic and genetic correlations as well as SNP-based heritability estimates between traits were calculated using the GCTA software [41]. For heritability estimation, a genomic relatedness matrix (GRM) was generated using SNPs located on the autosomes. The Restricted Maximum Likelihood (REML) analysis was then performed in GCTA using this GRM along with the phenotypic data. To control for fixed effects, the model included ranch, breed, sex, and age group, as well as the top five principal components from the multidimensional scaling (MDS) analysis. These covariates were incorporated using the *–covar* and *–qcovar* functions of the GCTA software. The statistical model can be represented as follows:

1 $$\begin{aligned} y = \alpha B + \beta W + \epsilon \end{aligned}$$

Where: y is the morphometric trait, B the vector of the fix factors (ranch, sex, age group, breed), W the vector of the additive genetic effect including the GRM, with a variance-covariance structure of

2 $$\begin{aligned} w \sim \ N\left( 0, G\sigma ^2_u\right) \end{aligned}$$

where G represents the genomic relationship matrix between individuals, $\sigma ^2_u$, the polygenic variance; and $\epsilon$ the vector of residual effects

3 $$\begin{aligned} \epsilon \sim N\left( 0,I\sigma ^2_e\right) \end{aligned}$$

I is an identity matrix of dimension (n x n) where n represents the sample size (856) and $\alpha$ and $\beta$ the incidence matrices for B and W respectively. The genetic correlation (r_g_) between pairs of body traits (x, y) was estimated in a bivariate genomic REML analyses and the phenotypic correlation (r_p_) between the two traits, derived from the bivariate genomic REML analysis output using the formula:

4 $$\begin{aligned} r_{\textrm{p}} = (\sigma w_{xy} + \sigma e_{xy})/\sqrt{\left( \sigma ^2_wx +\sigma ^2_ex\right) \left( \sigma ^2_wy + \sigma ^2_ey\right) } \end{aligned}$$

To estimate the standard error of the correlation, the Fisher’s z transformation was performed on the bivariate correlations (r_p_) following the formula:

5 $$\begin{aligned} Z = {1/2}ln ((1+r_{\textrm{p}})/(1-r_{\textrm{p}})) \end{aligned}$$

A standard error of Z is computed as:

6 $$\begin{aligned} SEz = 1\sqrt{N-3} \end{aligned}$$

The Z standard error is reverted back to correlation scale by:

7 $$\begin{aligned} SEr_{\textrm{p}} \approx SEz\sqrt{\left( 1-r_{\textrm{p}}^2\right) /(N-1)} \end{aligned}$$

### Estimation of linkage disequilibrium

Linkage disequilibrium between pairs of loci was measured for the Gudali and Simgud populations and the LD decay under four distance windows (< 100 kbp, 100 kbp to 500 kbp, 500 kbp to 1 mbp and >1 mbp) using TASSEL 5.2.13 software [42]. It was performed following Weir (Weir 1990) squared allele-frequency correlations (r^2^) which consider allele frequencies at loci. Fischer’s exact test [43] was used to calculate the LD estimate probabilities at least as extreme as those observed under a hypothesis of linkage equilibrium (*P*-values). LD decay as a function of distance between loci was computed by a non-linear regression model and the result was displayed as a plot in R version 4.1.2 software (Core Team).

### Genome-wide association analysis

Before performing the GWAS, we included the environmental and population stratification factors (MDS) as fixed effects by adding ranch, camp, herd, age, sex, and breed along with the top 20 MDS components as covariates in the model. The GWAS was performed to assess for any association between the measured body traits (BL, EL, HAW and SH) and molecular markers, using TASSEL 5.2.13, and fit in the association analysis with the Mixed Linear Model (MLM). The potential effect of the SNP markers on body traits was estimated following the model:

8 $$\begin{aligned} y = X\beta + Z\mu + \epsilon \end{aligned}$$

Where y is the vector of the observation (BL, SH, HAW, EL). $\beta$ represents the vector of fixed effect including SNP markers, ranch, camp, herd, age, sex, and breed, the kinship genetic matrix computed with the scaled-IBS method of PLINK, and the 20 first MDS principal components as covariates. $\mu$ is the vector of random additive genetic effects from various QTL for individuals SNPs and $\epsilon$ represents the vector of the residual effect value. The $\mu$ and $\epsilon$ are assumed to be following a normal distribution with mean of 0 and a variance equal $\sigma ^2\_a$. X, Z are the incidence matrices of $\beta$ and $\mu$ respectively. The results were displayed in the form of Manhattan and Quantile Quantile (Q-Q) plot in R using the qqman package [44] and ggplot2 [45]. To enhance the statistical power for the detection of associations, we have performed haplotype inference through imputation to higher-density SNP data [46, 47]. Genome imputation improves the resolution power for detecting association signals and uncovering novel variants [48]. We refitted the GWAS model with the same parameters but applied it to the genome imputed with Beagle v5.2 [49]. For the imputation, we used the largest available dataset of African indigenous cattle breeds as reference. This dataset includes 1082 animals from more than 30 local breeds, genotyped with the Illumina® BovineHD DNA Analysis Kit (Illumina, San Diego, CA), comprising approximately 777,962 SNPs, reported in [50]. Using a multibreed reference population has been shown to improve imputation accuracy [51]. Imputation resulted in 232,441 SNPs when considering only markers with Dosage R square (DR^2^) higher than 0.4. DR^2^ statistic is a Beagle internal estimator of imputation accuracy and a reliable proxy for selecting highly accurate imputed sites for downstream analyses [52]. Previous studies have suggested that DR^2^ values between 0.3 and 0.8 are acceptable thresholds for filtering [53]. After filtering, we achieved an average imputation accuracy of 0.64. For multiple testing correction, we applied the Bonferroni correction test with significance thresholds set as $\alpha =0.05$ and $\alpha =0.01$, and we defined the genome-wide significance threshold at ($p < 10$^−8^).

### Candidate gene identification and enrichment analysis

Markers showing significant associations with traits were used to query the Bovine ARS-UCD1.3 genome build via the NCBI Genome Data Viewer (https://www.ncbi.nlm.nih.gov/gdv/browser/genome/ accessed in May 2024), to locate potential candidate genes within a window of 100 kbp upstream and downstream of each SNP. The window was determined based on the extent of the average LD half-distance observed throughout the genome, to allow accurate identification of candidate genes. The location of genes and overlapping QTL from the Ensembl *Bos taurus* UMD1.3 assembly was determined using Biomart tool 2.62.0 [54]. Further we mapped the significant SNPs to the Animal QTL Database (https://www.animalgenome.org/cgi-bin/QTLdb/BT/index, accessed on 5 February 2025) to find if a significant region is a novel or existing QTL. Moreover, we performed enrichment and pathway analysis using the graphical web application ShinyGO 0.77 [55], accessing the KEGG database for enrichment analysis.

## Discussion

### Genetic correlation and heritability

The SNP-based estimated heritabilities of the four body measurements found in our study are considered moderate, as reported in previous studies [2, 14, 56, 57]. However, our heritability estimates are lower compared to those estimated for body traits in Benin local breeds [31] and in Wagyu cattle of China [3]. Genetic correlations found in our study show similar trends to the phenotypic correlations obtained. All the body traits considered were positively correlated and this interplay implies the potential to improve body traits together as a whole. Similar positive correlations were observed between production and body depth (0.138–0.228) in German Holstein [14]. Also, our genetic correlations are comparable to the results obtained in other cattle breeds, such as the local breeds of Benin [31], the Italian Jersey [58] and the Chinese Holstein [57]. The phenotypic correlation between HAW and SH in our study is similar to the 0.89 obtained in local breed of Benin by [31], though that study observed smaller correlations between other traits. Our significantly higher sample size would have improved the accuracy of the estimates.

### Population structure and linkage disequilibrium

Population stratification is one of the most common causes of false positive results in GWAS [59]. Without controlling for population structure, we only found (result not shown) a total of 30 SNPs significantly associated (*p* < E^−25^) with sacrum height; 27 SNPs significantly (*p* < E^−8^) associated with body length; 1 SNP significantly associated (*p* < E^−8^) with ear length and 1 with height at wither. Controlling for the structure of the population in our analysis has therefore improved the accuracy of the GWAS result. The MLM tends to be the model preferred for GWAS analysis since it controls for the stratification by integrating population structure, kinship, and family structure in the analysis [60]. Moreover, ancient and recent stratification of the population, as well as natural or artificial selection [61, 62], creates non-random associations (i.e. linkage disequilibrium) between alleles at different loci. Measuring these associations in our study, we found the maximum LD = 0.45, 0.15, 0.092, 0.056, and 0.037 at marker distances of 100, 250, 500, 1000, and >1000 kbp respectively. The result is similar to the average LD observed in Charolais, Limousin and Blonde d’Aquitaine beef breeds of France [63], which varied between 0.5 at distances smaller than 15 kbp, to less than 0.1 at distances greater than 120 kbp. Our LD estimates are however higher than those reported in most bovine studies which typically show average LD value close to zero for distances greater than 500 kbp. In the case of the Hawai’i cattle population, [64] found r^2^ of 0.15 reached at a distance of 100 kbp. Similarly, our results are higher than the r^2^ values of $\sim$ 0.35, 0.25, 0.22, 0.14, and 0.06 observed at marker distances 10, 20, 40, 100, and 1000 kbp, on Dutch and Australian Holstein–Friesian bulls, Australian Angus, New Zealand Friesian and Jersey cows respectively [7]. They are also higher than LD values ranging from 0.05 to 0.02 observed in the East African Zebu [30]. A decrease in LD with increasing marker distance is commonly observed in cattle [63, 65] with the decline occurring more rapidly in composite and crossbreed animals [66–68] as a consequence of breed formation and population history, such as bottleneck events [69].

One of the most interesting findings in our study is the existence of long range linkage disequilibrium (LRLD) in the genome of the Gudali and Simgud cattle, extending over distance greater than 1 mbp. The admixed genetic background of the Gudali and Simgud may explain the LRLD pattern observed. In fact, LRLD can result from admixture [70, 71], genetic drifts or epistatic selection [72] or chromosomal variations [73]. As cattle have been heavily selected, these long-range LD blocks are likely genuine. Although not yet extensively studied, these large stretches of LD reveal population specific patterns in human studies [74] and are only beginning to be investigated in cattle. Currently, the potential functional interactions between regions exhibiting LRLD remain elusive [63, 71]. It is usual within a breed to find SNPs associated with a QTL located hundreds of kilobases or megabases distant because of the persistence of substantial linkage disequilibrium [75]. While intense selection might explain the LRLD observed in the Simgud genome, the Gudali population might have undergone a population bottleneck with the intensive use of artificial insemination since it has been implicated in breeding initiatives with various Europrean taurine [37] and recently with the Simmental breed. Similar effects of bottlenecks in producing LRLD were observed in Blonde d’Aquitaine under intensive artificial selection [76].

### Genome-wide association analysis

By performing the first GWAS for body traits using autosomal SNPs on Cameroon indigenous Gudali cattle breed and its crossbred with Italian Simmental (Simgud), we identified 52 significantly associated variants, confirming the high complexity level of cattle genetic architecture of body traits [77, 78].

Body measures can be considered as indicators of animal condition in terms of health, immune response, welfare, and longevity [8, 9]. Identified genes or genomic regions should be targeted for any future cattle genomic selection in Cameroon.

The observed 52 SNPs were mapped to 70 genes including *CALN1, CNTNAP5, PTGDR2, TMEM109, TMEM132A, ADGRD1, ITGA10, NDUFAF1, NUSAP1, KCNA10, CYM, PROK1, LAMTOR5, SLC15A3, CCDC178, CELF4, SCNN1B, SCNN1G, NOS1AP, UHMK1* and *UNC5D* The consistent sample size used in our study improves the accuracy of our identified markers, which is crucial in association analysis [79].

Although further functional validation experiments in a different population could allow us to consolidate our studies, the biological function of some of our associated genes (e.g. *CNTNAP5, PTGDR2, UHMK1, ARHGEF10L*) - combined with HD genotype imputation which is known to improve the power of association analysis [80–82] and reveals additional candidate genes - make our study a strong initial baseline for further association analysis in Cameroon. Some of the QTL identified showed strong association with more than one of the body traits studied. For instance, on BTA10, the SNP ARS-BFGL-NGS-531 located at 36,829,871bp was associated with body length and height at wither. This locus was mapped to a genomic region harboring several genes including *NDUFAF1, CHP1, OIP5, NUSAP1, RTF1, ITPKA*. These genes were proposed as a candidate gene influencing inter-calving period in the Vrindavani cattle breed of India [83]. Another SNP associated with several traits is BovineHD1700013218 on BTA17:46,179,818 bp, which maps to *ADGRD1*. This marker showed association to all traits considered except sacrum height. The *ADGRD1* gene has been suggested as a potential candidate for carcass traits, mainly carcass weight, in Simmental beef cattle of China [84]. It encodes a protein that affects fatty acid concentration in chicken meat [85] and milk-related traits in Egyptian Buffalo, mainly fat and protein yields [86]. In humans, variations in the *ADGRD1* sequence were associated with metabolism, human height and heart frequency [87]. It is also associated with both human and mouse body weight [88]. The involvement of *ADGRD1* in lipid metabolism in different species suggests that it is a strong candidate gene for determining body size and growth. Some of the SNPs associated with more than one trait, such as BovineHD0100015405, BTA-81825-no-rs and BovineHD0600022959, were not mapped to any known QTL. These markers deserve further investigation, perhaps expanding the SNP windows beyond 100 kbp. The identification of these pleiotropic genes in our study confirms the high genetic correlation that was observed among all the traits considered and implies that body measures could be selected together for faster genetic improvement in Cameroon.

#### Candidate genes for sacrum height

The genome-wide analysis identified 33 SNPs associated with sacrum height and mapped within or close to 52 genes throughout the cattle genome. The vast majority of the identified genes were reported in previous studies as related to growth, feed intake, immune response, reproduction and fertility and carcass traits in various cattle breeds worldwide. On BTA2, the SNP BovineHD0200039720 was mapped to Rho Guanine Nucleotide Exchange Factor 10 Like (*ARHGEF10L*) gene. The exact role of this gene in growth has not yet been elucidated. Interestingly, another member of the same family, *ARHGEF2*, has been linked to childhood obesity in humans [89] as well as to intramuscular fatty acid composition in pigs [90]. Other members of the ARGHGEF family have been associated with cattle omental fat (*ARHGEF5* [91]), resistance to disease and bacterial infection [92] and gastrointestinal parasite resistance in Spanish sheep [93] for *ARHGEF17*. Based on these findings, we speculated that *ARHGEF10L* might contribute to higher body size in cattle and should be considered as a candidate gene for body size and growth in cattle. The strongest associations were found on BTA3 with SNPs BovineHD0300010326 and BovineHD0300010324 (found only 2.4 kbp apart). This genomic region harbors five candidate genes, namely *KCNA10, CYM, PROK1, LAMTOR5 and RBM15*. None of these identified genes were previously reported as directly associated with sacrum height. However, the Late Endosomal/Lysosomal Adaptor, MAPK and MTOR Activator 5 (*LAMTOR5*) gene was found to be associated with beef cattle growth traits and reproductive traits [94]. Moreover, *LAMTOR5* was identified as a candidate gene for weight gain in both Hereford and Bradford beef cattle [95]. It is also implicated in the immune response through the regulation of Mammalian Toll-like receptors [96], which play a role in the defense mechanism against pathogens [97]. By participating in pathogen response, *LAMTOR5* contributes to maintaining cattle in good health and therefore ensuring optimal growth and body size. *KCNA10* was proposed as a candidate gene associated with beef production and carcass quality traits in Chikso and Hanwoo cattle [98]. *CYM* was previously reported as an immunity-related gene and also associated with milk fat percentage in South African cattle [12] and fatty acid composition in Chinese Wagyu cattle [99]. Likewise, *RBM15* was revealed as a potential candidate gene for clinical mastitis resistance [100]. These genes, located in a known body size-related QTL should be considered potential candidates for sacrum height in Gudali and Simgud. The SNPs Hapmap51849-BTA-68314 and BovineHD0300020629 found on BTA3 at positions 69,713,184 bp and 69,700,962 bp respectively were mapped near the LIM Homeobox8 (*LHX8*) gene known for its association with oocyte development [101]. It encodes a specific transcription factor, essential for postnatal folliculogenesis. In an association study in Nellore cattle [102], *LHX8* was suggested to be associated with calving interval. The participation of *LHX8* in early development makes it a potential candidate for body size in cattle. For the SNP BovineHD0300006766 on BTA3 associated with sacrum height and the corresponding gene Integrin alpha 10 (*ITGA10*), there is no prior reported association with cattle body traits. However, *ITGA10* is generally suggested to be crucial in cell adhesion and migration, as well as the regulation of the inflammatory response [103]. It is implicated in several vital processes in cattle: in particular, genome-wide mRNA and miRNA expression analysis in Nellore cattle [104] linked *ITGA10* with mineral concentrations in muscle. Mineral balance and composition in cells affect almost all physiological processes and in bovines can affect growth, health, reproduction as well as meat quality. This implies that *ITGA10* might participate in the body size of cattle through the effect of mineral amount and composition. The genomic region around *ITGA10* harbors eleven genes including *TXNIP* which was previously reported as a candidate gene for glucose metabolism in mid-lactation Holstein [105]. On the same chromosome, at position 101,700,489 bp, the SNP ARS-BFGL-NGS-2973 falls within the *ERI3* gene - previously linked to metabolic body weight in mid-lactation Holstein [105]. The SNP BovineHD1200011574 on BTA12: 40,470,693 bp and associated gene *PCDH9* was previously associated with fat deposition and backfat thickness [105, 107] and therefore represents a candidate gene for body size because of its role in lipid metabolism. On BTA24, six important candidate genes were found and related to body size (*CCDC178*), marbling (*SMAD7*), immunity (*PHLPP1*), stress tolerance (*BCL2*), and two novel candidate genes *CELF4* and *CTIF*. The *CCDC178* gene was associated with body size and especially birth weight in alpine cattle breed [108]. It was also identified in a GWAS study on hoof disorders in Austrian Fleckvieh and Braunvieh [109]. Another member of the same family (*CCDC117*) was found associated with feed intake and heat stress regulation in cattle [110, 111]. *SMAD7* is a transcription factor with potential relation to meat quality and especially marbling in cattle [112]. The *PHLPP1* (PH domain and leucine rich repeat protein phosphatase 1) gene was found as a candidate gene associated to gastrointestinal nematode resistance in German black pied cattle [113]. These parasites have major effects on pasture-grazed cattle, especially leading to decreases in milk production and female fertility [114–116]. The *BCL2* protein family regulates embryonic development and growth [117, 118] by creating a balance between its pro- and anti-apoptosis genes [119]. This gene is of paramount importance for embryonic development and animal growth in tropical environments filled with challenges such as heat stress, parasites, poor pasture and management. It was identified as a possible candidate for adaptive selection in North African cattle [120]. For its key role in embryo development and growth, *BCL2* could be considered a novel candidate gene for body size in cattle. We also identified two genes that have not previously been associated with cattle production traits: *CELF4* and *CTIF*. The *CELF4* gene encodes an RNA-binding protein, expressed mainly in the central nervous system, which is implicated in the regulation of several genes both co-transcriptionally and post-transcriptionally [121]. It was found in a genomic region displaying signatures of selection in North African cattle [120]. The *CTIF* gene is associated with longissimus muscle area, known to be a good indicator of growth and production [122, 123] in Nellore cattle [124]. Due to its association with growth-related indicators, *CTIF* should be considered a novel candidate gene for body size in cattle. On BTA25, the highly significant SNP ARS-BFGL-NGS-7030 was mapped close to sodium channel epithelial 1 subunit B and G (*SCNN1B, SCNN1G*). These are both part of the ENaC epithelial sodium concentration regulatory path - involved in salt taste and sodium ingestion [125]. Salt is also a major component of cattle saliva that helps in rough forage digestion. The salt content is directly linked to growth since it influences forage, water intake, dry matter digestibility and rumen fermentation [126], especially in beef cattle reared under poor quality forage. Therefore we concluded that *SCNN1B* and *SCNN1G* are strong candidate genes regulating body size in cattle. The SNP BovineHD2500010674 at 37,709,326 bp was mapped to *BAIAP2L1* and upstream of *BRI3, TECPR1, BHLHA15* and *LMTK2*. The Brain-specific angiogenesis inhibitor 1 (BAI1)-associated protein 2-like 1 (*BAIAP2L1*) is involved in plasma membrane protrusion and actin formation during cell morphogenesis and migration [127]. It was identified as a candidate gene for volatile fatty acid production in a GWAS of ruminant methane emission using Holstein cattle [128]. By acting on actin formation it is clearly participating in growth and development of the animals. Therefore we speculate that *BAIAP2L1* might be active in cattle body size through maintaining cell shape and polarity. The *BHLHA15* gene plays an important role in growth and development through its critical role in embryogenesis especially in gastrulae and plantule stages in mouse [129]. By participating in embryo development, the*BHLHA15* contributes to body size and should therefore be consider candidate gene for growth and body size in cattle. Another significant association on BTA25 was the SNP BovineHD2500008133 and ARS-BFGL-NGS-101637, both within the *CALN1* gene. This gene was reported as potentially associated to longevity in Chinese Holsteins [130] and feed efficiency in mid-lactation Holsteins [131]. It was also associated with lipid absorption/metabolism in Duroc, Landrace and Yorkshire pigs [96]. By playing role in feed efficiency and lipid metabolism, *CALN1* directly participates in growth and body size and should therefore be considered candidate gene for improvement of growth in cattle. Finally, the significantly associated SNP ARS-BFGL-NGS- 78220 is located within the *TMEM248* gene, previously proposed as a candidate for feed intake in mid-lactation Holstein [131]. On BTA27, SNP ARS-BFGL-NGS-107550 was mapped to the *UNC5D* gene which is within a known QTL for body depth, calving ease, stature, feet and leg conformation. It is also linked to residual feed intake in mid-lactation Hosltein [105].

#### Candidate genes for body length

Beside the pleiotropic genes discussed previously, the genome-wide association identified 13 SNPs significantly related to body length. Two SNPs were found on BTA2 at positions 76,976,290 bp and 76,839,374 bp, both within the *CNTNAP5* gene, which has been previously associated with differences in hip cross height in Brahman versus Yunling cattle [132]. This gene also showed significant association with bicostal diameter in Sudanese goats [133]. A partial deletion in the *NRXN1*, a homologous gene of *CNTNAP5*, resulted in short stature in humans [134], reinforcing the hypothesis of a possible link between the *CNTNAP5* locus and animal stature. A variant on BTA29 (ARS-BFGL-NGS-15883) at position 37,283,656 bp, was located within *SLC15A3* and near the *PTGDR2, PRPF19, TMEM109, TMEM132A* and *CD6* genes. The *SLC15A3* gene belongs to the solute carrier gene family (SLC) protein which is known as the largest set of cell transporters for nutrients such as sugars, SCFAs and amino acids [135, 136]. Members of the SLC family generally participate in growth and adaptation. For instance, a polymorphism at the 5’ UTR of *SLC44A5* was found to be associated with birth weight in Holsteins and thus could be considered to control dystocia in cattle [137]. *SLC16A4* is within a known QTL related to body length in cattle. It was recently associated to feed efficiency in indigenous cattle breeds of Benin [31]. More recently [105], *SLC45A2* was associated to heat tolerance in a genome-wide association analysis of milk production in Thai dairy cattle. Likewise, a polymorphism in *SLC11A1* was associated to bovine tuberculosis (bTB) resistance [56] while *SLC6A6* was suggested to be associated with bTB resistance in Irish Holsteins [138]. This gene family has also been associated with ribeye area in Nellore cattle [106]. Because *SLC16A4* has been already associated with body length, feed efficiency and belongs to the SLC family which transport nutrients to cells thus providing the necessary energy for basic metabolism and growth, we concluded that *SLC15A3* is a strong candidate gene for BL in cattle and should be considered for cattle breeding in a tropical environments like Cameroon characterized by heat stress and parasite constraints. The *PTGDR2* is a heat stress related gene implicated in thermoregulation [139] and fever response [140]. its expression is increased during heat shock and it regulates vasodilation as a key response to reduce temperature through a gradient from from the skin to the ambient air. Temperature above 25^o^C implies limited activities due to thermal stress and subsequent decline in rate of dry matter intake from grazing animals [141] which might lead to reduced body weight and size. Although this gene has not been associated with body traits, its potential effects on dry matter intake during heat stress condition that is frequent in tropical conditions, make PTGDR2 a candidate gene for body traits in Gudali and Simgud. The same region also harbors two transmembrane protein genes: *TMEM109* and *TMEM132A*. Transmembrane proteins constitute a large family of genes participating in various processes such as male fertility and growth [142] in Chinese indigenous cattle breeds, intramuscular fat content in Nellore breed [83, 143], childhood and adult obesity in humans [144, 145] and in immune response [146]. For their implication in growth, immunity and lipid metabolism, we speculated that *TMEM109* and *TMEM132A* are candidate genes for the body length in Gudali and Simgud cattle breeds.

#### Candidate genes for ear length

The five genome-wide significant SNPs identified were mapped to six genes. The SNP BTA-69126-no-rs on BTA3 is located in a region harbouring the *UHMK1, SH2D1B, NOS1AP* and *SPATA46* genes. Among these genes, *UHMK1* and *NOS1AP* were linked to saturated fatty acid profile in intramuscular fat of the longissimus thoracis muscle of Nellore cattle [147]. Moreover, *NOS1AP* showed direct association with body size, especially chest width, in Xinjiang Brown cattle [148]. Therefore we speculate that *NOS1AP* and *UHMK1* are novel candidate genes for ear length in Gudali and Simgud cattle because of their participation in body conformation and lipid metabolism in cattle. On BTA27, a SNP at position 16,529,725 bp was mapped within *FAT1*, a candidate gene for reproductive traits in Holstein [149].

#### Candidate genes for height at wither

The six genome-wide significant SNPs found related to height at wither were mapped to 7 candidate genes. The BovineHD1700013218 and ARS-BFGL-NGS-531 were located in a genomic region exhibiting pleiotropic effect and have been discussed earlier.

#### Candidate genes from imputation analysis

In addition to the above mentioned body trait genes identified, using the imputed genome provided more potential associations, with genes such as *COLEC12, GNAI3, ATXN7L2, COMMD10, GRM8, DAB1, USP8, CCDC83* and *USP24*. *COLEC12* is within a QTL related to feed conversion ratio and residual feed intake, parameters known to affect feed efficiency which is directly correlated with animal growth [150]. Therefore this gene/QTL should be considered a candidate for selecting for body size in cattle. *GNAI3* is involved in various cellular processes, including proliferation, apoptosis, cytokinesis, and differentiation [151, 152]. It was reported to be related directly to body conformation traits in Korean Holstein Population [153]. Moreover, *GNAI3* has a potential implication in heat tolerance mechanisms in goat [154]. It represents a promising candidate gene for body size in cattle in a tropical context like Cameroon. *ATXN7L2* is another candidate gene regulating skeletal muscle development. Although it has not been directly associated with body size in prior studies, its known function makes it worth investigating as a strong candidate for body size. *COMMD10* plays a role in various tissues, being involved in ubiquitin expression. It was recently revealed to play a new and critical role in neural development [155]. Although no previous association was reported between GRM8 and the body traits studied here, this gene was proposed as a candidate for chest circumference in Brahman and Yunling cattle breeds [132]. The imputation, by increasing the number and density of SNP available for association analysis [156], improved the resolution of marker-trait association [157]. Moreover, using a large multi-breed composite imputation reference panel is known to further improve imputation accuracy compared to within breed reference panel [51]. We obtained stronger associations, with higher *P*-values than for the genotype for all traits. The enrichment analysis shows that our candidate genes mostly participate in ubiquitin-mediated proteolysis, sumoylation, sodium intake and taste transduction pathways. It is reported that high sodium intake for pregnant cows is associated with increased birth weight of their calves [158]. Similarly, taste plays a major role in food intake [159] and in maintaining a healthy diet [160]. Meanwhile ubiquitination and sumoylation are both crucial for immunity, disease resistance and inflammatory response [161, 162], all of which are crucial for animal adaptation and growth, particularly in harsh tropical environments such as those found in Cameroon. Our study has identified key candidate genes that could be targeted for genetic selection in Gudali and Simgud cattle of Cameroon. As such, it provides a valuable resource to inform decision-making aimed at improving growth performance in these cattle populations in Cameroon. Additionally, we have explored the enriched pathways associated with these candidate genes to gain deeper insight into the genetic basis of body traits in Gudali and Simgud.

## Conclusion

This study represents the first genome-wide analysis of the local zebu Gudali and its crossbred with the Italian Simmental (Simgud) in semi-extensive system in Cameroon. The study revealed moderate heritability, positive genetic and phenotypic correlation among the four traits. Using a Mixed Linear Model approach, we have produced a comprehensive candidate gene set associated with major body traits in the population. Although these associations have not yet been validated in a broader population, many of the identified genes and genomic regions align with findings from previous studies, thereby strengthening confidence in the validity of the associations. These associations will however be confirmed in future studies with the breeders at SODEPA to improve cattle production in Cameroon. Furthermore, we identified several novel candidate genes associated to reproduction, growth, disease resistance traits. Given their known biological functions, these candidate genes could be valuable targets for genetic improvement efforts in Gudali and Simgud cattle. Our work thus represents a considerable resource for the foundation of a genomic breeding programme in Gudali and the Simgud crossbreed.

## Supplementary information

Additional file 1. Map of the sampling zone.

Additional file 2. Photograph of a Gudali animal.

Additional file 3. Photograph of a Simgud animal.

Additional file 4. Manhattan plots of the body traits using imputation to higher density SNP dataset.

Additional file 5. KEGG enrichment of candidate genes on BTA3 and BTA25.

Additional file 6. Aldosterone-regulated sodium reabsorption (a) and taste transduction (b) pathways.

Additional file 7. Phenotypic distribution and ANOVA table.

Additional file 8. MDS result of the 20 components included as covariates in the GWAS.

Additional file 9. Novel candidate genes associated with body traits based on imputation data.

Additional file 10. Phenotypic data used for the association analysis.

## Data Availability

The datasets generated and analysed during the current study are available at European Variant Archive (https://www.ebi.ac.uk/eva/) as project PRJEB79966 and analysis ERZ24835341. The phenotypic data used in this study is provided in supplementary informations.

## References

[1] Gritsenko S, Ruchay A, Kolpakov V, Lebedev S, Guo H, Pezzuolo A. On-barn forecasting beef cattle production based on automated non-contact body measurement system. Animals. 2023;13(4):611. 10.3390/ani13040611 PMC9951648 36830398

[2] Munim T, Oikawa T, Ibi T, Kunieda T. Genetic relationship of body measurement traits at early age with carcass traits in Japanese black cattle. Anim Sci J. 2013;84(3):206–12. 23480700 10.1111/asj.12005

[3] An B, Xia J, Chang T, Wang X, Xu L, Zhang L, et al. Genome-wide association study reveals candidate genes associated with body measurement traits in Chinese Wagyu beef cattle. Anim Genet. 2019;50(4):386–90. 31179577 10.1111/age.12805

[4] Pryce JE, Hayes BJ, Bolormaa S, Goddard ME. Polymorphic regions affecting human height also control stature in cattle. Genetics. 2011;187(3):981–4. 21212230 10.1534/genetics.110.123943 PMC3048786

[5] Cole JB, Wiggans GR, Ma L, Sonstegard TS, Lawlor TJ, Crooker BA, et al. Genome-wide association analysis of thirty one production, health, reproduction and body conformation traits in contemporary US Holstein cows. BMC Genomics. 2011;12:1–17. 10.1186/1471-2164-12-408 PMC3176260 21831322

[6] Lee SH, Choi BH, Lim D, Gondro C, Cho YM, Dang CG, et al. Genome-wide association study identifies major loci for carcass weight on BTA14 in Hanwoo (Korean cattle). PLoS ONE. 2013;8(10):e74677. 24116007 10.1371/journal.pone.0074677 PMC3792095

[7] de Oliveira Bessa AF, Duarte INH, Rola LD, Bernardes PA, Neto SG, Lôbo RB, et al. Genetic evaluation for reproductive and productive traits in Brahman cattle. Theriogenology. 2021;173:261–8. 34403971 10.1016/j.theriogenology.2021.08.008

[8] Vollema A, Van Der Beek S, Harbers A, De Jong G. Genetic evaluation for longevity of Dutch dairy bulls. J Dairy Sci. 2000;83(11):2629–39. 11104283 10.3168/jds.S0022-0302(00)75156-3

[9] Dadpasand M, Miraei-Ashtiani S, Shahrebabak MM, Torshizi RV. Impact of conformation traits on functional longevity of Holstein cattle of Iran assessed by a Weibull proportional hazards model. Livest Sci. 2008;118(3):204–11.

[10] Forabosco F, Groen A, Bozzi R, Van Arendonk J, Filippini F, Boettcher P, et al. Phenotypic relationships between longevity, type traits, and production in Chianina beef cattle. J Anim Sci. 2004;82(6):1572–80. 15216982 10.2527/2004.8261572x

[11] Setati M, Norris D, Banga C, Benyi K. Relationships between longevity and linear type traits in Holstein cattle population of Southern Africa. Trop Anim Health Prod. 2004;36:807–14. 15643816 10.1023/b:trop.0000045965.99974.9c

[12] Matika O, Riggio V, Anselme-Moizan M, Law AS, Pong-Wong R, Archibald AL, et al. Genome-wide association reveals QTL for growth, bone and in vivo carcass traits as assessed by computed tomography in Scottish Blackface lambs. Genet Sel Evol. 2016;48:1–15. 26856324 10.1186/s12711-016-0191-3 PMC4745175

[13] Naserkheil M, Lee DH, Mehrban H. Improving the accuracy of genomic evaluation for linear body measurement traits using single-step genomic best linear unbiased prediction in Hanwoo beef cattle. BMC Genetics. 2020;21:1–9. 33267771 10.1186/s12863-020-00928-1 PMC7709290

[14] Schmidtmann C, Segelke D, Bennewitz J, Tetens J, Thaller G. Genetic analysis of production traits and body size measurements and their relationships with metabolic diseases in German Holstein cattle. J Dairy Sci. 2023;106(1):421–38. 36424319 10.3168/jds.2022-22363

[15] Dawson CR, Henley PA, Schroeder AR, Meteer WT, Hayes CA, Felix TL, et al. Effects of rubber matting on feedlot cattle growth performance, locomotion, and carcass characteristics in slatted floor facilities. J Anim Sci. 2022;100(3):skac041. 35148402 10.1093/jas/skac041 PMC9030117

[16] Larson HE, Jaderborg JP, Paulus-Compart DM, Crawford GI, DiCostanzo A. Effect of substitution of distillers grains and glycerin for steam–flaked corn in finishing cattle diets on growth performance and carcass characteristics. J Anim Sci. 2023;101:skac348. 36592746 10.1093/jas/skac348 PMC9831090

[17] Ring SC, Graham DA, Kelleher MM, Doherty ML, Berry DP. Genetic parameters for variability in the birth of persistently infected cattle following likely in utero exposure to bovine viral diarrhea virus. J Anim Sci. 2019;97(2):559–68. 30412254 10.1093/jas/sky430 PMC6358239

[18] Kambal S, Tijjani A, Ibrahim SA, Ahmed MKA, Mwacharo JM, Hanotte O. Candidate signatures of positive selection for environmental adaptation in indigenous African cattle: A review. Anim Genet. 2023;54(6):689–708. 37697736 10.1111/age.13353

[19] Weerasinghe W. The accuracy and bias of estimates of breed composition and inference about genetic structure using high density SNP markers in Australian sheep breeds. Armidale: University of New England; 2014.

[20] Werner F, Durstewitz G, Habermann FA, Thaller G, Krämer W, Kollers S, et al. Detection and characterization of SNPs useful for identity control and parentage testing in major European dairy breeds. Anim Genet. 2004;35(1):44–9. 14731229 10.1046/j.1365-2052.2003.01071.x

[21] McTavish EJ, Hillis DM. A genomic approach for distinguishing between recent and ancient admixture as applied to cattle. J Hered. 2014;105(4):445–56. 24510946 10.1093/jhered/esu001 PMC4048551

[22] Pant SD, Schenkel FS, Verschoor CP, You Q, Kelton DF, Moore SS, et al. A principal component regression based genome wide analysis approach reveals the presence of a novel QTL on BTA7 for MAP resistance in Holstein cattle. Genomics. 2010;95(3):176–82. 20060464 10.1016/j.ygeno.2010.01.001

[23] Barendse W, Harrison BE, Bunch RJ, Thomas MB, Turner LB. Genome wide signatures of positive selection: the comparison of independent samples and the identification of regions associated to traits. BMC Genomics. 2009;10:1–15. 19393047 10.1186/1471-2164-10-178 PMC2681478

[24] Wiggans G, VanRaden P, Cooper T. The genomic evaluation system in the United States: Past, present, future. J Dairy Sci. 2011;94(6):3202–11. 21605789 10.3168/jds.2010-3866

[25] MacArthur J, Bowler E, Cerezo M, Gil L, Hall P, Hastings E, et al. The new NHGRI-EBI Catalog of published genome-wide association studies (GWAS Catalog). Nucleic Acids Res. 2017;45(D1):D896–901. 27899670 10.1093/nar/gkw1133 PMC5210590

[26] Hirschhorn JN, Daly MJ. Genome-wide association studies for common diseases and complex traits. Nat Rev Genet. 2005;6(2):95–108. 15716906 10.1038/nrg1521

[27] Strucken EM, Al-Mamun HA, Esquivelzeta-Rabell C, Gondro C, Mwai OA, Gibson JP. Genetic tests for estimating dairy breed proportion and parentage assignment in East African crossbred cattle. Genet Sel Evol. 2017;49:1–18. 28899355 10.1186/s12711-017-0342-1 PMC5596489

[28] Gebrehiwot NZ, Strucken EM, Marshall K, Aliloo H, Gibson JP. SNP panels for the estimation of dairy breed proportion and parentage assignment in African crossbred dairy cattle. Genet Sel Evol. 2021;53:1–18. 33653262 10.1186/s12711-021-00615-4 PMC7923343

[29] Gautier M, Flori L, Riebler A, Jaffrézic F, Laloé D, Gut I, et al. A whole genome Bayesian scan for adaptive genetic divergence in West African cattle. BMC Genomics. 2009;10:1–18. 19930592 10.1186/1471-2164-10-550 PMC2784811

[30] Mbole-Kariuki MN, Sonstegard T, Orth A, Thumbi S, Bronsvoort BdC, Kiara H, et al. Genome-wide analysis reveals the ancient and recent admixture history of East African Shorthorn Zebu from Western Kenya. Heredity. 2014;113(4):297–305. 24736786 10.1038/hdy.2014.31 PMC4181064

[31] Vanvanhossou SFU, Scheper C, Dossa LH, Yin T, Brügemann K, König S. A multi-breed GWAS for morphometric traits in four Beninese indigenous cattle breeds reveals loci associated with conformation, carcass and adaptive traits. BMC Genomics. 2020;21:1–16. 10.1186/s12864-020-07170-0 PMC7656759 33176675

[32] Ema PN, Manjeli Y, Meutchieyié F, Keambou C, Wanjala B, Desta A, et al. Genetic diversity of four Cameroonian indigenous cattle using microsatellite markers. J Livest Sci. 2014;5:9–17.

[33] Paguem A, Abanda B, Achukwi MD, Baskaran P, Czemmel S, Renz A, et al. Whole genome characterization of autochthonous Bos taurus brachyceros and introduced Bos indicus indicus cattle breeds in Cameroon regarding their adaptive phenotypic traits and pathogen resistance. BMC Genet. 2020;21:1–15. 32571206 10.1186/s12863-020-00869-9 PMC7309992

[34] Tawah C, Rege J. Gudali cattle of west and central africa. Anim Genet Resour. 1996;17:147–64.

[35] Djoko TD, Mbah DA, Mbanya JN, Kamga P, Awah NR, Bopelet M. Crossbreeding cattle for milk production in the tropics: effects of genetic and environmental factors on the performance of improved genotypes on the Cameroon Western high plateau= Cruces de ganado para la produccion lactea en los tropicos: efectos de los factores genéticos y ambientales en el rendimiento de los genotipos mejorados en las mesetas atlas de Camerun del Oeste= Croisement des bovins pour la production laitière sous les tropiques: effets des facteurs génétiques et environnementaux sur les performances des génotypes améliorés des hauts-plateaux de l'Ouest Cameroun. Rev Elev Med Vet Pays Trop. 2003;56(1-2).

[36] Tawah C, Mbah D, Rege J, Oumate H. Genetic evaluation of birth and weaning weight of Gudali and two-breed synthetic Wakwa beef cattle populations under selection in Cameroon: genetic and phenotypic parameters. Anim Sci. 1993;57(1):73–9.

[37] Lhoste P. Cattle breeds of Adamawa (Cameroon). In: Colloque sur I’élevage. Fort-Larny; 1969. pp. 519–533.

[38] Guha P, Das A, Dutta S, Chaudhuri TK. A rapid and efficient DNA extraction protocol from fresh and frozen human blood samples. J Clin Lab Anal. 2018;32(1):e22181. 28233351 10.1002/jcla.22181 PMC6817243

[39] Purcell S, Neale B, Todd-Brown K, Thomas L, Ferreira MA, Bender D, et al. PLINK: a tool set for whole-genome association and population-based linkage analyses. Am J Hum Genet. 2007;81(3):559–75. 17701901 10.1086/519795 PMC1950838

[40] Sempéré G, Moazami-Goudarzi K, Eggen A, Laloë D, Gautier M, Flori L. WIDDE: a Web-Interfaced next generation database for genetic diversity exploration, with a first application in cattle. BMC Genomics. 2015;16(1):940. 26573482 10.1186/s12864-015-2181-1 PMC4647285

[41] Yang J, Lee SH, Goddard ME, Visscher PM. GCTA: a tool for genome-wide complex trait analysis. Am J Hum Genet. 2011;88(1):76–82. 21167468 10.1016/j.ajhg.2010.11.011 PMC3014363

[42] Bradbury PJ, Zhang Z, Kroon DE, Casstevens TM, Ramdoss Y, Buckler ES. TASSEL: software for association mapping of complex traits in diverse samples. Bioinformatics. 2007;23(19):2633–5. 17586829 10.1093/bioinformatics/btm308

[43] Fisher RA. The logic of inductive inference. J R Stat Soc. 1935;98(1):39–82.

[44] Turner SD. qqman: an R package for visualizing GWAS results using QQ and manhattan plots. J Open Source Softw. 2018;3:731.

[45] Wickham H. ggplot2: Elegant Graphics for Data Analysis. New York: Springer-Verlag; 2016.

[46] Marchini J, Howie B, Myers S, McVean G, Donnelly P. A new multipoint method for genome-wide association studies by imputation of genotypes. Nat Genet. 2007;39(7):906–13. 17572673 10.1038/ng2088

[47] Spencer CC, Su Z, Donnelly P, Marchini J. Designing genome-wide association studies: sample size, power, imputation, and the choice of genotyping chip. PLoS Genet. 2009;5(5):e1000477. 19492015 10.1371/journal.pgen.1000477 PMC2688469

[48] Porcu E, Sanna S, Fuchsberger C, Fritsche LG. Genotype imputation in genome-wide association studies. Curr Protoc Hum Genet. 2013;78(1):1–25. 10.1002/0471142905.hg0125s78 23853078

[49] Browning BL, Zhou Y, Browning SR. A one-penny imputed genome from next-generation reference panels. Am J Hum Genet. 2018;103(3):338–48. 30100085 10.1016/j.ajhg.2018.07.015 PMC6128308

[50] Tijjani A, Kambal S, Terefe E, Njeru R, Ogugo M, Ndambuki G, et al. Genomic Reference Resource for African Cattle: Genome Sequences and High-Density Array Variants. Sci Data. 2024;11(1):801. 39030190 10.1038/s41597-024-03589-2 PMC11271538

[51] Rowan TN, Hoff JL, Crum TE, Taylor JF, Schnabel RD, Decker JE. A multi-breed reference panel and additional rare variants maximize imputation accuracy in cattle. Genet Sel Evol. 2019;51:1–16. 31878893 10.1186/s12711-019-0519-x PMC6933688

[52] Dorji J, Chamberlain AJ, Reich CM, VanderJagt CJ, Nguyen TV, Daetwyler HD, et al. Mitochondrial sequence variants: testing imputation accuracy and their association with dairy cattle milk traits. Genet Sel Evol. 2024;56(1):62. 39266998 10.1186/s12711-024-00931-5 PMC11391750

[53] Browning BL, Browning SR. A unified approach to genotype imputation and haplotype-phase inference for large data sets of trios and unrelated individuals. Am J Hum Genet. 2009;84(2):210–23. 19200528 10.1016/j.ajhg.2009.01.005 PMC2668004

[54] Durinck S, Moreau Y, Kasprzyk A, Davis S, De Moor B, Brazma A, et al. BioMart and Bioconductor: a powerful link between biological databases and microarray data analysis. Bioinformatics. 2005;21(16):3439–40. 16082012 10.1093/bioinformatics/bti525

[55] Ge SX, Jung D, Yao R. ShinyGO: a graphical gene-set enrichment tool for animals and plants. Bioinformatics. 2020;36(8):2628–9. 31882993 10.1093/bioinformatics/btz931 PMC7178415

[56] Kadarmideen H, Ali A, Thomson P, Müller B, Zinsstag J. Polymorphisms of the SLC11A1 gene and resistance to bovine tuberculosis in African Zebu cattle. Anim Genet. 2011;42(6):656–8. 22035008 10.1111/j.1365-2052.2011.02203.x

[57] Zhang X, Chu Q, Guo G, Dong G, Li X, Zhang Q, et al. Genome-wide association studies identified multiple genetic loci for body size at four growth stages in Chinese Holstein cattle. PLoS ONE. 2017;12(4):e0175971. 28426785 10.1371/journal.pone.0175971 PMC5398616

[58] Roveglia C, Niero G, Bobbo T, Penasa M, Finocchiaro R, Visentin G, et al. Genetic parameters for linear type traits including locomotion in Italian Jersey cattle breed. Livest Sci. 2019;229:131–6.

[59] Hellwege JN, Keaton JM, Giri A, Gao X, Velez Edwards DR, Edwards TL. Population stratification in genetic association studies. Curr Protoc Hum Genet. 2017;95(1):1–22. 10.1002/cphg.48 PMC6007879 29044472

[60] Yu J, Pressoir G, Briggs WH, Vroh Bi I, Yamasaki M, Doebley JF, et al. A unified mixed-model method for association mapping that accounts for multiple levels of relatedness. Nat Genet. 2006;38(2):203–8. 16380716 10.1038/ng1702

[61] Ardlie KG, Kruglyak L, Seielstad M. Patterns of linkage disequilibrium in the human genome. Nat Rev Genet. 2002;3(4):299–309. 11967554 10.1038/nrg777

[62] Qanbari S, Pimentel E, Tetens J, Thaller G, Lichtner P, Sharifi AR, et al. The pattern of linkage disequilibrium in German Holstein cattle. Anim Genet. 2010;41(4):346–56. 20055813 10.1111/j.1365-2052.2009.02011.x

[63] El Hou A, Rocha D, Venot E, Blanquet V, Philippe R. Long-range linkage disequilibrium in French beef cattle breeds. Genet Sel Evol. 2021;53:1–14. 34301193 10.1186/s12711-021-00657-8 PMC8306006

[64] Adhikari M, Kantar MB, Longman RJ, Lee C, Oshiro M, Caires K, et al. Genome-wide association study for carcass weight in pasture-finished beef cattle in Hawai’i. Front Genet. 2023;14:1168150. 37229195 10.3389/fgene.2023.1168150 PMC10203587

[65] Kumar H, Panigrahi M, Saravanan K, Parida S, Bhushan B, Gaur G, et al. SNPs with intermediate minor allele frequencies facilitate accurate breed assignment of Indian Tharparkar cattle. Gene. 2021;777:145473. 33549713 10.1016/j.gene.2021.145473

[66] Deng T, Liang A, Liu J, Hua G, Ye T, Liu S, et al. Genome-wide SNP data revealed the extent of linkage disequilibrium, persistence of phase and effective population size in purebred and crossbred buffalo populations. Front Genet. 2019;9:688. 30671082 10.3389/fgene.2018.00688 PMC6332145

[67] Prieur V, Clarke SM, Brito LF, McEwan JC, Lee MA, Brauning R, et al. Estimation of linkage disequilibrium and effective population size in New Zealand sheep using three different methods to create genetic maps. BMC Genet. 2017;18:1–19. 28732466 10.1186/s12863-017-0534-2 PMC5521107

[68] Mulim HA, Brito LF, Batista Pinto LF, Moletta JL, Da Silva LR, Pedrosa VB. Genetic and genomic characterization of a new beef cattle composite breed (Purunã) developed for production in pasture-based systems. Front Genet. 2022;13:858970. 35923708 10.3389/fgene.2022.858970 PMC9341487

[69] Brito LF, Jafarikia M, Grossi DA, Kijas JW, Porto-Neto LR, Ventura RV, et al. Characterization of linkage disequilibrium, consistency of gametic phase and admixture in Australian and Canadian goats. BMC Genet. 2015;16:1–15. 26108536 10.1186/s12863-015-0220-1 PMC4479065

[70] Wilson JF, Goldstein DB. Consistent long-range linkage disequilibrium generated by admixture in a Bantu-Semitic hybrid population. Am J Hum Genet. 2000;67(4):926–35. 10961910 10.1086/303083 PMC1287894

[71] Bahbahani H. Long-range linkage disequilibrium events on the genome of dromedary camels as a signal of epistatic and directional positive selection. Heliyon. 2024;10(14). Elsevier. 10.1016/j.heliyon.2024.e34343 PMC11295981 39100441

[72] Id-Lahoucine S, Molina A, Cánovas A, Casellas J. Screening for epistatic selection signatures: a simulation study. Sci Rep. 2019;9(1):1026. 30705409 10.1038/s41598-019-38689-2 PMC6355851

[73] Cáceres A, Sindi SS, Raphael BJ, Cáceres M, González JR. Identification of polymorphic inversions from genotypes. BMC Bioinformatics. 2012;13:1–16. 22321652 10.1186/1471-2105-13-28 PMC3296650

[74] Park L. Population-specific long-range linkage disequilibrium in the human genome and its influence on identifying common disease variants. Sci Rep. 2019;9(1):11380. 31388069 10.1038/s41598-019-47832-y PMC6684625

[75] Bolormaa S, Pryce J, Hayes B, Goddard M. Multivariate analysis of a genome-wide association study in dairy cattle. J Dairy Sci. 2010;93(8):3818–33. 20655452 10.3168/jds.2009-2980

[76] Bouquet A, Renand G, Phocas F. Evolution de la diversité génétique des populations françaises de bovins allaitants spécialisés de 1979 à 2008. INRA Prod Anim. 2009;22(4):317–30.

[77] Bouwman AC, Daetwyler HD, Chamberlain AJ, Ponce CH, Sargolzaei M, Schenkel FS, et al. Meta-analysis of genome-wide association studies for cattle stature identifies common genes that regulate body size in mammals. Nat Genet. 2018;50(3):362–7. 29459679 10.1038/s41588-018-0056-5

[78] Doyle JL, Berry DP, Veerkamp RF, Carthy TR, Walsh SW, Evans RD, et al. Genomic regions associated with skeletal type traits in beef and dairy cattle are common to regions associated with carcass traits, feed intake and calving difficulty. Front Genet. 2020;11:20. 32117439 10.3389/fgene.2020.00020 PMC7010604

[79] Goddard ME, Hayes BJ. Mapping genes for complex traits in domestic animals and their use in breeding programmes. Nat Rev Genet. 2009;10(6):381–91. 19448663 10.1038/nrg2575

[80] MacLeod I, Bowman P, Vander Jagt C, Haile-Mariam M, Kemper K, Chamberlain A, et al. Exploiting biological priors and sequence variants enhances QTL discovery and genomic prediction of complex traits. BMC Genomics. 2016;17:1–21. 26920147 10.1186/s12864-016-2443-6 PMC4769584

[81] Erbe M, Hayes B, Matukumalli L, Goswami S, Bowman P, Reich C, et al. Improving accuracy of genomic predictions within and between dairy cattle breeds with imputed high-density single nucleotide polymorphism panels. J Dairy Sci. 2012;95(7):4114–29. 22720968 10.3168/jds.2011-5019

[82] Fang L, Sahana G, Ma P, Su G, Yu Y, Zhang S, et al. Use of biological priors enhances understanding of genetic architecture and genomic prediction of complex traits within and between dairy cattle breeds. BMC Genomics. 2017;18:1–12. 28797230 10.1186/s12864-017-4004-z PMC5553760

[83] Gangwar M, Kumar S, Ahmad SF, Singh A, Agrawal S, Anitta P, et al. Identification of genetic variants affecting reproduction traits in Vrindavani cattle. Mamm Genome. 2024;35(1):99–111. 37924370 10.1007/s00335-023-10023-2

[84] Wang X, Miao J, Chang T, Xia J, An B, Li Y, et al. Evaluation of GBLUP, BayesB and elastic net for genomic prediction in Chinese Simmental beef cattle. PLoS ONE. 2019;14(2):e0210442. 30817758 10.1371/journal.pone.0210442 PMC6394919

[85] Yang S, Wang Y, Wang L, Shi Z, Ou X, Wu D, et al. RNA-Seq reveals differentially expressed genes affecting polyunsaturated fatty acids percentage in the Huangshan Black chicken population. PLoS ONE. 2018;13(4):e0195132. 29672513 10.1371/journal.pone.0195132 PMC5908183

[86] Abdel-Shafy H, Awad MA, El-Regalaty H, El-Assal SD, Abou-Bakr S. Prospecting genomic regions associated with milk production traits in Egyptian buffalo. J Dairy Res. 2020;87(4):389–96. 33185171 10.1017/S0022029920000953

[87] Fischer L, Wilde C, Schöneberg T, Liebscher I. Functional relevance of naturally occurring mutations in adhesion G protein-coupled receptor ADGRD1 (GPR133). BMC Genomics. 2016;17:1–9. 27516204 10.1186/s12864-016-2937-2 PMC4982218

[88] Mansego ML, Milagro FI, Zulet MÁ, Moreno-Aliaga MJ, Martínez JA. Differential DNA methylation in relation to age and health risks of obesity. Int J Mol Sci. 2015;16(8):16816–32. 26213922 10.3390/ijms160816816 PMC4581172

[89] Zhu ZL, Yang QM, Li C, Chen J, Xiang M, Chen MM, et al. Identification of biomarkers for childhood obesity based on expressional correlation and functional similarity. Mol Med Rep. 2018;17(1):109–16. 29115457 10.3892/mmr.2017.7913 PMC5780071

[90] Ramayo-Caldas Y, Ballester M, Fortes MR, Esteve-Codina A, Castelló A, Noguera JL, et al. From SNP co-association to RNA co-expression: Novel insights into gene networks for intramuscular fatty acid composition in porcine. BMC Genomics. 2014;15:1–15. 24666776 10.1186/1471-2164-15-232 PMC3987146

[91] Pan C, Yang C, Wang S, Ma Y. Identifying key genes and functionally enriched pathways of diverse adipose tissue types in cattle. Front Genet. 2022;13:790690. 35237299 10.3389/fgene.2022.790690 PMC8884536

[92] Ghoreishifar SM, Eriksson S, Johansson AM, Khansefid M, Moghaddaszadeh-Ahrabi S, Parna N, et al. Signatures of selection reveal candidate genes involved in economic traits and cold acclimation in five Swedish cattle breeds. Genet Sel Evol. 2020;52:1–15. 32887549 10.1186/s12711-020-00571-5 PMC7487911

[93] Atlija M, Arranz JJ, Martinez-Valladares M, Gutiérrez-Gil B. Detection and replication of QTL underlying resistance to gastrointestinal nematodes in adult sheep using the ovine 50K SNP array. Genet Sel Evol. 2016;48:1–16. 26791855 10.1186/s12711-016-0182-4 PMC4719203

[94] Ribeiro VMP, Gouveia GC, de Moraes MM, de Araújo AEM, Raidan FSS, de Souza Fonseca PA, et al. Genes underlying genetic correlation between growth, reproductive and parasite burden traits in beef cattle. Livest Sci. 2021;244:104332.

[95] Campos GS, Sollero BP, Reimann FA, Junqueira VS, Cardoso LL, Yokoo MJI, et al. Tag-SNP selection using Bayesian genomewide association study for growth traits in Hereford and Braford cattle. J Anim Breeding Genet. 2020;137(5):449–67. 10.1111/jbg.12458 31777136

[96] Zhang W, Zhuang N, Liu X, He L, He Y, Mahinthichaichan P, et al. The metabolic regulator Lamtor5 suppresses inflammatory signaling via regulating mTOR-mediated TLR4 degradation. Cell Mol Immunol. 2020;17(10):1063–76. 31467416 10.1038/s41423-019-0281-6 PMC7608472

[97] Kawai T, Akira S. The role of pattern-recognition receptors in innate immunity: update on Toll-like receptors. Nat Immunol. 2010;11(5):373–84. 20404851 10.1038/ni.1863

[98] Edea Z, Jung KS, Shin SS, Yoo SW, Choi JW, Kim KS. Signatures of positive selection underlying beef production traits in Korean cattle breeds. J Anim Sci Technol. 2020;62(3):293. 32568261 10.5187/jast.2020.62.3.293 PMC7288235

[99] Wang Z, Zhu B, Niu H, Zhang W, Xu L, Xu L, et al. Genome wide association study identifies SNPs associated with fatty acid composition in Chinese Wagyu cattle. J Anim Sci Biotechnol. 2019;10:1–13. 30867906 10.1186/s40104-019-0322-0 PMC6399853

[100] Cai Z, Iso-Touru T, Sanchez MP, Kadri N, Bouwman AC, Chitneedi PK, et al. Meta-analysis of six dairy cattle breeds reveals biologically relevant candidate genes for mastitis resistance. Genet Sel Evol. 2024;56(1):54. 39009986 10.1186/s12711-024-00920-8 PMC11247842

[101] Fu L, Zhang M, Mastrantoni K, Perfetto M, Wei S, Yao J. Bovine Lhx8, a germ cell-specific nuclear factor, interacts with Figla. PLoS ONE. 2016;11(10):e0164671. 27716808 10.1371/journal.pone.0164671 PMC5055334

[102] Diaz-Miranda EA, Penitente-Filho JM, Gomez-Leon VE, Neto TM, Guimarães SF, Siqueira JB, Guimarães JD. Selection based on the Breeding Soundness Evaluation is associated with the improvement of the reproductive quality of young Nellore bulls. Theriogenology. 2024;226;369–77. Elsevier. 10.1016/j.theriogenology.2024.06.032 38970923

[103] Lemma SA, Kuusisto M, Haapasaari KM, Sormunen R, Lehtinen T, Klaavuniemi T, et al. Integrin alpha 10, CD44, PTEN, cadherin-11 and lactoferrin expressions are potential biomarkers for selecting patients in need of central nervous system prophylaxis in diffuse large B-cell lymphoma. Carcinogenesis. 2017;38(8):812–20. 28854563 10.1093/carcin/bgx061 PMC5862348

[104] Afonso J, Fortes MRS, Reverter A, Diniz WJdS, Cesar ASM, Lima AOd, et al. Genetic regulators of mineral amount in Nelore cattle muscle predicted by a new co-expression and regulatory impact factor approach. Sci Rep. 2020;10(1):8436. 10.1038/s41598-020-65454-7 PMC7242321 32439843

[105] Buaban S, Lengnudum K, Boonkum W, Phakdeedindan P. Genome-wide association study on milk production and somatic cell score for Thai dairy cattle using weighted single-step approach with random regression test-day model. J Dairy Sci. 2022;105(1):468–94. 34756438 10.3168/jds.2020-19826

[106] Júnior GF, Costa R, De Camargo G, Carvalheiro R, Rosa G, Baldi F, et al. Genome scan for postmortem carcass traits in Nellore cattle. J Anim Sci. 2016;94(10):4087–95. 27898882 10.2527/jas.2016-0632

[107] Seong J, Yoon H, Kong HS. Identification of microRNA and target gene associated with marbling score in Korean cattle (Hanwoo). Genes Genomics. 2016;38:529–38.

[108] Mancin E, Tuliozi B, Pegolo S, Sartori C, Mantovani R. Genome wide association study of beef traits in local Alpine breed reveals the diversity of the pathways involved and the role of time stratification. Front Genet. 2022;12:746665. 35058966 10.3389/fgene.2021.746665 PMC8764395

[109] Kosińska-Selbi B, Suchocki T, Egger-Danner C, Schwarzenbacher H, Frąszczak M, Szyda J. Exploring the potential genetic heterogeneity in the incidence of hoof disorders in Austrian Fleckvieh and Braunvieh cattle. Front Genet. 2020;11:577116. 33281874 10.3389/fgene.2020.577116 PMC7705352

[110] Kubik RM. Genomic investigation of beta agonist supplementation and heat stress in livestock species. 2018.

[111] Lindholm-Perry A, Butler A, Kern R, Hill R, Kuehn L, Wells J, et al. Differential gene expression in the duodenum, jejunum and ileum among crossbred beef steers with divergent gain and feed intake phenotypes. Anim Genet. 2016;47(4):408–27. 27226174 10.1111/age.12440

[112] Cassar-Malek I, Pomiès L, De La Foye A, Tournayre J, Boby C, Hocquette JF. Transcriptome profiling reveals stress-responsive gene networks in cattle muscles. PeerJ. 2022;10:e13150. 35411255 10.7717/peerj.13150 PMC8994496

[113] May K, Scheper C, Brügemann K, Yin T, Strube C, Korkuć P, et al. Genome-wide associations and functional gene analyses for endoparasite resistance in an endangered population of native German Black Pied cattle. BMC Genomics. 2019;20:1–15. 30961534 10.1186/s12864-019-5659-4 PMC6454736

[114] Charlier J, Claerebout E, Duchateau L, Vercruysse J. A survey to determine relationships between bulk tank milk antibodies against Ostertagia ostertagi and milk production parameters. Vet Parasitol. 2005;129(1–2):67–75. 15817205 10.1016/j.vetpar.2004.11.024

[115] Mezo M, González-Warleta M, Castro-Hermida JA, Muiño L, Ubeira FM. Association between anti-*F. hepatica* antibody levels in milk and production losses in dairy cows. Vet Parasitol. 2011;180(3-4):237–42. 10.1016/j.vetpar.2011.03.009 21459514

[116] Charlier J, Duchateau L, Claerebout E, Williams D, Vercruysse J. Associations between anti-Fasciola hepatica antibody levels in bulk-tank milk samples and production parameters in dairy herds. Prev Vet Med. 2007;78(1):57–66. 17095109 10.1016/j.prevetmed.2006.09.010

[117] Matwee C, Betts DH, King WA. Apoptosis in the early bovine embryo. Zygote. 2000;8(1):57–68. 10840875 10.1017/s0967199400000836

[118] Yang M, Rajamahendran R. Involvement of apoptosis in bovine blastocysts produced in vitro. Theriogenology. 1999;51(1):336.

[119] Mirkes PE. 2001 Warkany lecture: to die or not to die, the role of apoptosis in normal and abnormal mammalian development. Teratology. 2002;65(5):228–39. 11967922 10.1002/tera.10049

[120] Ben-Jemaa S, Mastrangelo S, Lee SH, Lee JH, Boussaha M. Genome-wide scan for selection signatures reveals novel insights into the adaptive capacity in local North African cattle. Sci Rep. 2020;10(1):19466. 33173134 10.1038/s41598-020-76576-3 PMC7655849

[121] Wagnon JL, Briese M, Sun W, Mahaffey CL, Curk T, Rot G, et al. CELF4 regulates translation and local abundance of a vast set of mRNAs, including genes associated with regulation of synaptic function. PLoS Genet. 2012;8(11):e1003067. 23209433 10.1371/journal.pgen.1003067 PMC3510034

[122] Boggs DL, Merkel RA. Live animal carcass evaluation and selection manual. 1993. https://agris.fao.org/search/en/providers/123819/records/647363d153aa8c89630bd713.

[123] Bergen R, Miller S, Wilton J. Genetic correlations among indicator traits for carcass composition measured in yearling beef bulls and finished feedlot steers. Can J Anim Sci. 2005;85(4):463–73.

[124] Medeiros de Oliveira Silva R, Bonvino Stafuzza N, de Oliveira Fragomeni B, Miguel Ferreira de Camargo G, Matos Ceacero T, Noely dos Santos Gonçalves Cyrillo J, et al. Genome-wide association study for carcass traits in an experimental Nelore cattle population. PLoS ONE. 2017;12(1):e0169860. 10.1371/journal.pone.0169860 PMC5261778 28118362

[125] Chandrashekar J, Kuhn C, Oka Y, Yarmolinsky DA, Hummler E, Ryba NJ, et al. The cells and peripheral representation of sodium taste in mice. Nature. 2010;464(7286):297–301. 20107438 10.1038/nature08783 PMC2849629

[126] White HC, Davis NG, Van Emon ML, Wyffels SA, DelCurto T. Impacts of increasing levels of salt on intake, digestion, and rumen fermentation with beef cattle consuming low-quality forages. Transl Anim Sci. 2019;3(Supplement_1):1818–21. 10.1093/tas/txz111 PMC6999140 32704960

[127] Chao A, Tsai CL, Jung SM, Chuang WC, Kao C, Hsu A, et al. BAI1-associated protein 2-like 1 (BAIAP2L1) is a potential biomarker in ovarian cancer. PLoS ONE. 2015;10(7):e0133081. 26222696 10.1371/journal.pone.0133081 PMC4519316

[128] Jalil Sarghale A, Moradi Shahrebabak M, Moradi Shahrebabak H, Nejati Javaremi A, Saatchi M, Khansefid M, et al. Genome-wide association studies for methane emission and ruminal volatile fatty acids using Holstein cattle sequence data. BMC Genet. 2020;21:1–14. 33228565 10.1186/s12863-020-00953-0 PMC7684878

[129] Pin CL, Bonvissuto AC, Konieczny SF. Mist1 expression is a common link among serous exocrine cells exhibiting regulated exocytosis. Anat Rec: Off Publ Am Assoc Anatomists. 2000;259(2):157–67. 10.1002/(SICI)1097-0185(20000601)259:2<157::AID-AR6>3.0.CO;2-0 10820318

[130] Zhang H, Liu A, Wang Y, Luo H, Yan X, Guo X, et al. Genetic parameters and genome-wide association studies of eight longevity traits representing either full or partial lifespan in Chinese Holsteins. Front Genet. 2021;12:634986. 33719343 10.3389/fgene.2021.634986 PMC7947242

[131] Hardie L, VandeHaar M, Tempelman R, Weigel K, Armentano L, Wiggans G, et al. The genetic and biological basis of feed efficiency in mid-lactation Holstein dairy cows. J Dairy Sci. 2017;100(11):9061–75. 28843688 10.3168/jds.2017-12604

[132] Chen Q, Huang B, Zhan J, Wang J, Qu K, Zhang F, et al. Whole-genome analyses identify loci and selective signals associated with body size in cattle. J Anim Sci. 2020;98(3):skaa068. 10.1093/jas/skaa068 PMC7097718 32115622

[133] Rahmatalla S, Arends D, Reissmann M, Wimmers K, Reyer H, Brockmann G. Genome-wide association study of body morphological traits in Sudanese goats. Anim Genet. 2018;49(5):478–82. 30062755 10.1111/age.12686

[134] Bermudez-Wagner K, Jeng LJ, Slavotinek AM, Sanford EF. 2p16. 3 microdeletion with partial deletion of the neurexin-1 gene in a female with developmental delays, short stature, and a congenital diaphragmatic hernia. Clin Dysmorphol. 2013;22(1):22–4. 10.1097/MCD.0b013e32835b8df2 23207424

[135] Gao S, Gong G, Wang X, Gao X, Guo X, Luo Y, et al. Classification of SLC family-related genes involved in ferroptosis predicts lung cancer prognosis and immunotherapy response. Sci Rep. 2023;13(1):20032. 37973895 10.1038/s41598-023-47328-w PMC10654497

[136] Cesar-Razquin A, Snijder B, Frappier-Brinton T, Isserlin R, Gyimesi G, Bai X, et al. A call for systematic research on solute carriers. Cell. 2015;162(3):478–87. 26232220 10.1016/j.cell.2015.07.022

[137] Sugimoto M, Watanabe T, Sugimoto Y. The molecular effects of a polymorphism in the 5prime UTR of solute carrier family 44, member 5 that is associated with birth weight in Holsteins. PLoS ONE. 2012;7(7):e41267. 22815983 10.1371/journal.pone.0041267 PMC3399839

[138] Finlay EK, Berry DP, Wickham B, Gormley EP, Bradley DG. A genome wide association scan of bovine tuberculosis susceptibility in Holstein-Friesian dairy cattle. PLoS ONE. 2012;7(2):e30545. 22355315 10.1371/journal.pone.0030545 PMC3280253

[139] Garner J, Chamberlain A, Vander Jagt C, Nguyen T, Mason B, Marett L, et al. Gene expression of the heat stress response in bovine peripheral white blood cells and milk somatic cells in vivo. Sci Rep. 2020;10(1):19181. 33154392 10.1038/s41598-020-75438-2 PMC7645416

[140] Gao W, Schmidtko A, Wobst I, Lu R, Angioni C, Geisslinger G. Prostaglandin D2 produced by hematopoietic prostaglandin D synthase contributes to LPS-induced fever. J Physiol Pharmacol. 2009;60:145–50. 19617658

[141] National Research Council (US). Committee on Animal Nutrition. Nutrient requirements of domestic animals. National Academy Press; 1944.

[142] Guo X, Zhang S, Yang H, Pei J, Wu X, Bao P, et al. Bovine TMEM95 gene: Polymorphisms detecting in five Chinese indigenous cattle breeds and their association with growth traits. Electron J Biotechnol. 2021;51:58–66.

[143] Cesar AS, Regitano LC, Reecy JM, Poleti MD, Oliveira PS, de Oliveira GB, et al. Identification of putative regulatory regions and transcription factors associated with intramuscular fat content traits. BMC Genomics. 2018;19:1–20. 29945546 10.1186/s12864-018-4871-y PMC6020320

[144] Todendi PF, Klinger EI, Geraldo AC, Brixner L, Reuter CP, Lindenau JDR, et al. Genetic risk score based on fat mass and obesity-associated, transmembrane protein 18 and fibronectin type III domain containing 5 polymorphisms is associated with anthropometric characteristics in South Brazilian children and adolescents. Br J Nutr. 2019;121(1):93–9. 30311592 10.1017/S0007114518002738

[145] Ma M, Lee JH, Kim M. Identification of a TMEM182 rs141764639 polymorphism associated with central obesity by regulating tumor necrosis factor-[CDATA[\alpha]] in a Korean population. J Diabetes Complicat. 2020;34(12):107732. 10.1016/j.jdiacomp.2020.107732 32938560

[146] Schiavo G, Bertolini F, Utzeri VJ, Ribani A, Geraci C, Santoro L, et al. Taking advantage from phenotype variability in a local animal genetic resource: identification of genomic regions associated with the hairless phenotype in Casertana pigs. Anim Genet. 2018;49(4):321–5. 29672877 10.1111/age.12665

[147] Lemos MV, Chiaia HLJ, Berton MP, Feitosa FL, Aboujaoud C, Camargo GM, et al. Genome-wide association between single nucleotide polymorphisms with beef fatty acid profile in Nellore cattle using the single step procedure. BMC Genomics. 2016;17:1–16. 26960694 10.1186/s12864-016-2511-y PMC4784275

[148] Zhang M, Wang Y, Chen Q, Wang D, Zhang X, Huang X, et al. Genome-Wide Association Study on Body Conformation Traits in Xinjiang Brown Cattle. Int J Mol Sci. 2024;25(19):10557. 39408884 10.3390/ijms251910557 PMC11476655

[149] Seabury CM, Smith JL, Wilson ML, Bhattarai E, Santos JE, Chebel RC, et al. Genome-wide association and genomic prediction for a reproductive index summarizing fertility outcomes in US Holsteins. G3: Genes Genomes Genet. 2023;13(9):jkad043. 10.1093/g3journal/jkad043 PMC10468724 36848195

[150] Archer J, Richardson E, Herd R, Arthur P. Potential for selection to improve efficiency of feed use in beef cattle: a review. Aust J Agric Res. 1999;50(2):147–62.

[151] Martinez-Ceballos E, Chambon P, Gudas LJ. Differences in gene expression between wild type and Hoxa1 knockout embryonic stem cells after retinoic acid treatment or leukemia inhibitory factor (LIF) removal. J Biol Chem. 2005;280(16):16484–98. 15722554 10.1074/jbc.M414397200

[152] Chen X, Macica CM, Nasiri A, Broadus AE. Regulation of articular chondrocyte proliferation and differentiation by Indian hedgehog and parathyroid hormone-related protein in mice. Arthritis Rheum Off J Am Coll Rheumatol. 2008;58(12):3788–97. 10.1002/art.23985 PMC2599803 19035497

[153] Haque MA, Alam MZ, Iqbal A, Lee YM, Dang CG, Kim JJ. Genome-wide Association studies for body conformation traits in Korean Holstein Population. Animals. 2023;13(18):2964. 37760364 10.3390/ani13182964 PMC10526087

[154] Rangkasenee N, Murani E, Brunner R, Schellander K, Cinar MU, Scholz AM, et al. KRT8, FAF1 and PTH1R gene polymorphisms are associated with leg weakness traits in pigs. Mol Biol Rep. 2013;40:2859–66. 23196707 10.1007/s11033-012-2301-9

[155] Phan KP, Pelargos P, Tsytsykova AV, Tsitsikov EN, Wiley G, Li C, et al. COMMD10 Is Essential for Neural Plate Development during Embryogenesis. J Dev Biol. 2023;11(1):13. 36976102 10.3390/jdb11010013 PMC10051640

[156] Naj AC. Genotype imputation in genome-wide association studies. Curr Protoc Hum Genet. 2019;102(1):e84. 31216114 10.1002/cphg.84

[157] Howie BN, Donnelly P, Marchini J. A flexible and accurate genotype imputation method for the next generation of genome-wide association studies. PLoS Genet. 2009;5(6):e1000529. 19543373 10.1371/journal.pgen.1000529 PMC2689936

[158] Mohamed M, Phillips C. The effect of increasing the salt intake of pregnant dairy cows on the salt appetite and growth of their calves. Anim Sci. 2003;77(1):181–5.

[159] Ftuwi H, Parri R, Mohammed A. Novel, fully characterised bovine taste bud cells of fungiform papillae. Cells. 2021;10:2285. 34571933 10.3390/cells10092285 PMC8469975

[160] Peng Y, Gillis-Smith S, Jin H, Tränkner D, Ryba NJ, Zuker CS. Sweet and bitter taste in the brain of awake behaving animals. Nature. 2015;527(7579):512–5. 26580015 10.1038/nature15763 PMC4712381

[161] Ciechanover A, Orian A, Schwartz AL. Ubiquitin-mediated proteolysis: biological regulation via destruction. Bioessays. 2000;22(5):442–51. 10797484 10.1002/(SICI)1521-1878(200005)22:5<442::AID-BIES6>3.0.CO;2-Q

[162] Huang CH, Yang TT, Lin KI. Mechanisms and functions of SUMOylation in health and disease: a review focusing on immune cells. J Biomed Sci. 2024;31(1):16. 38280996 10.1186/s12929-024-01003-y PMC10821541

