# Supplementary figures and images for: Genome-wide association analysis revealed novel candidate genes for body measurement traits in indigenous Gudali and crossbred Simgud in Cameroon
Source: BMC Genomics. 2025 Jul 14;26:664. doi: 10.1186/s12864-025-11865-7 (PMC12257737; doi:10.1186/s12864-025-11865-7)

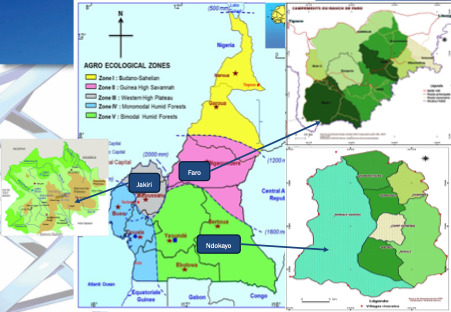

Supplement: Supplementary file 1 — Additional file 1. Map of the sampling zone. [file 12864_2025_11865_MOESM1_ESM.jpg]

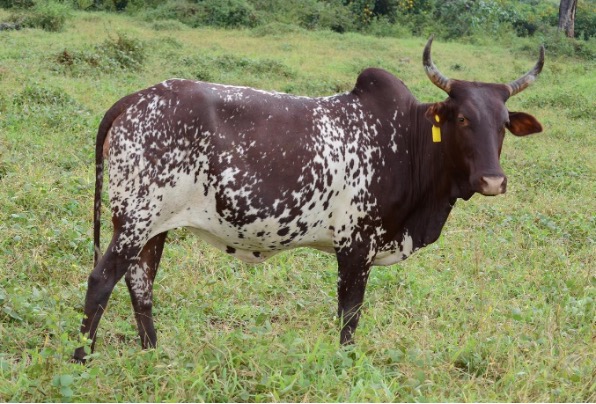

Supplement: Supplementary file 2 — Additional file 2. Photograph of a Gudali animal. [file 12864_2025_11865_MOESM2_ESM.jpg]

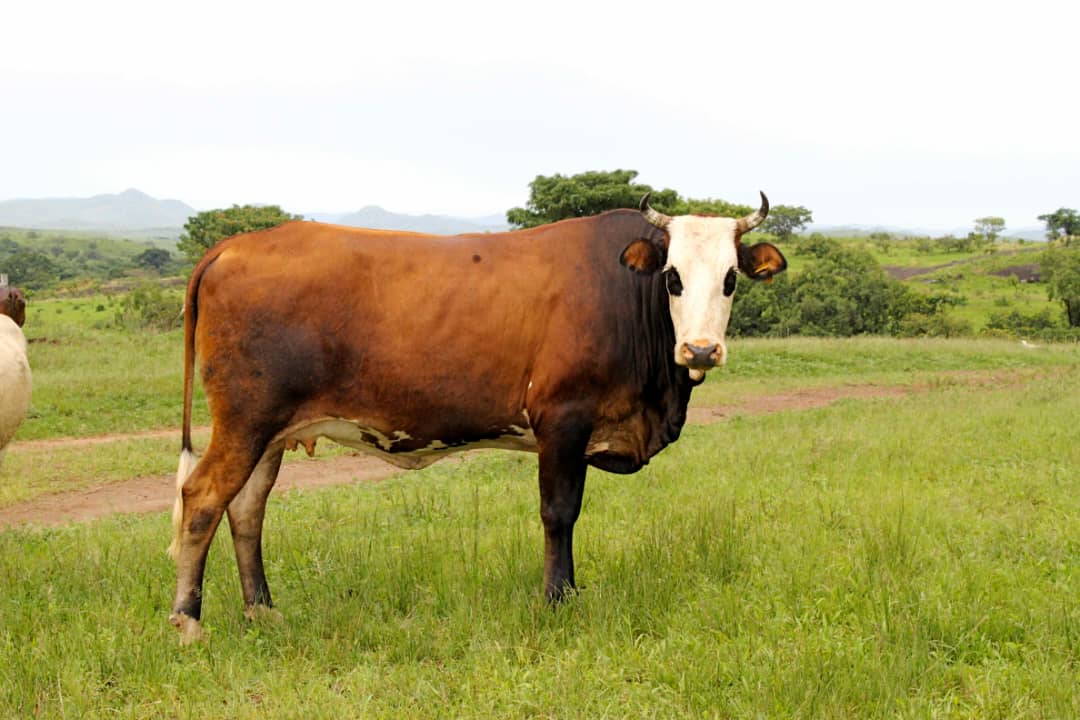

Supplement: Supplementary file 3 — Additional file 3. Photograph of a Simgud animal. [file 12864_2025_11865_MOESM3_ESM.jpeg]

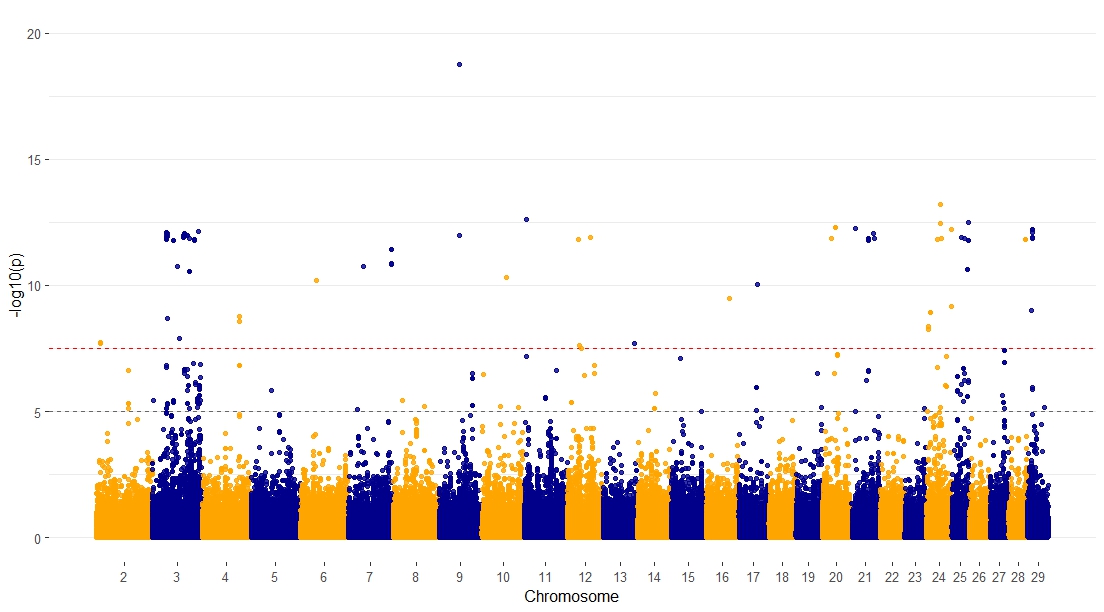

Supplement: Supplementary file 4 — Additional file 4. Manhattan plots of the body traits using imputation to higher density SNP dataset. [file 12864_2025_11865_MOESM4_ESM.zip › Suppl 4. Figure A4 1 Manhattan plot for SH using imputed genome.jpeg]

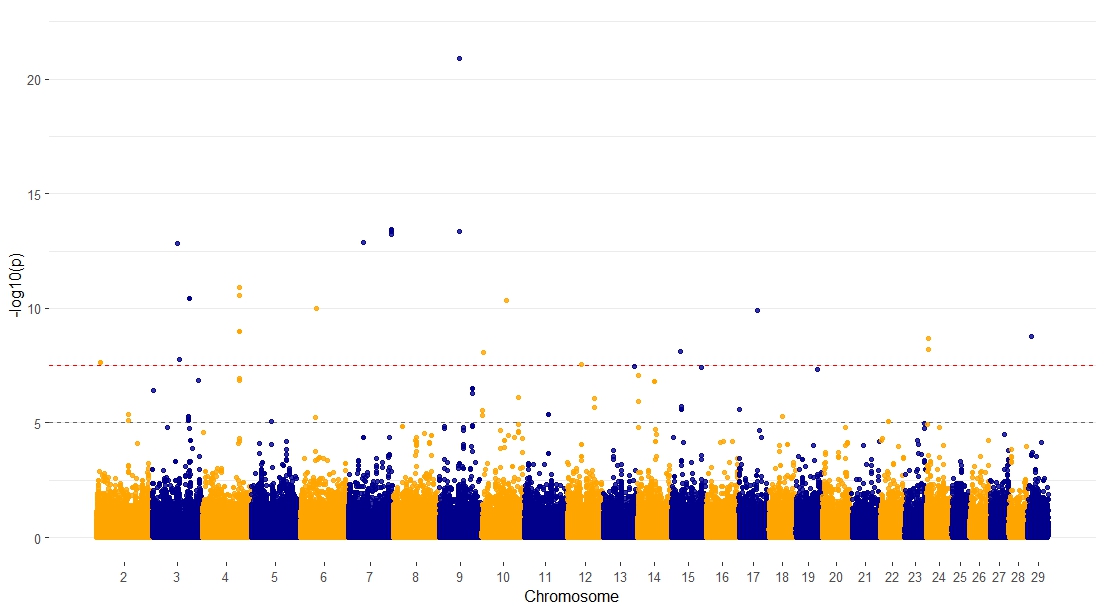

Supplement: Supplementary file 4 — Additional file 4. Manhattan plots of the body traits using imputation to higher density SNP dataset. [file 12864_2025_11865_MOESM4_ESM.zip › Suppl 4. Figure A4 3 Manhattan plot for HAW using imputed genome.jpeg]

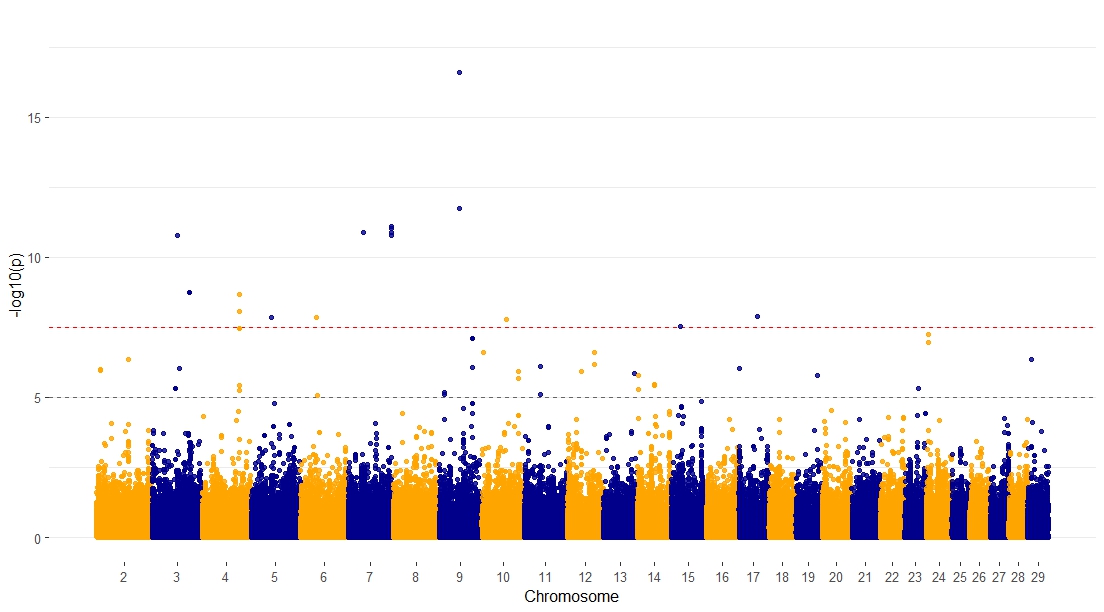

Supplement: Supplementary file 4 — Additional file 4. Manhattan plots of the body traits using imputation to higher density SNP dataset. [file 12864_2025_11865_MOESM4_ESM.zip › Suppl. 4 . Figure A4 2 Manhattan plot for BL using imputed genome.jpeg]

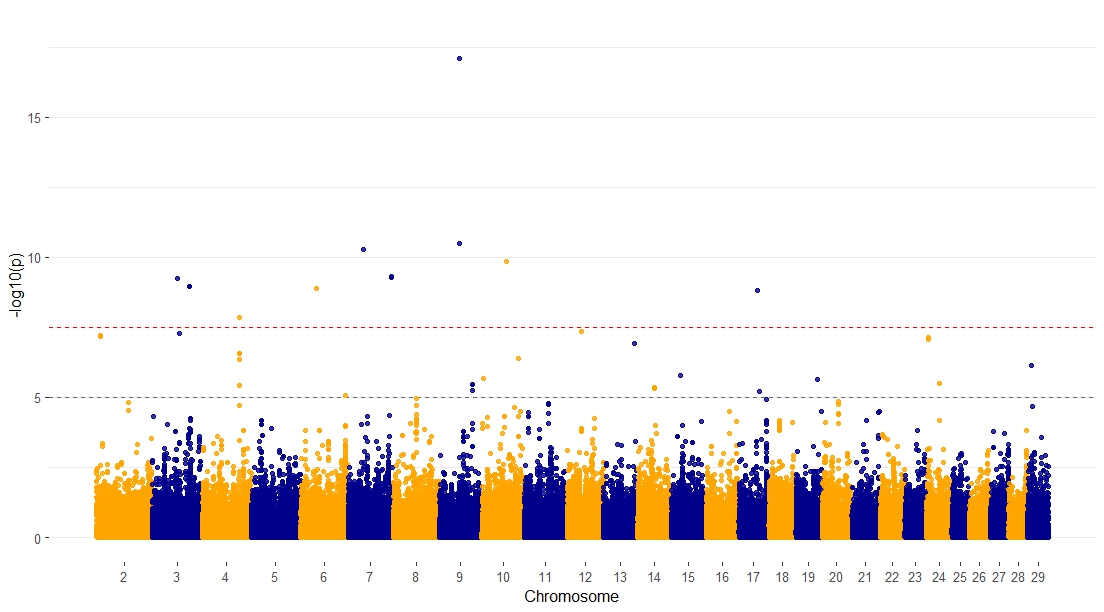

Supplement: Supplementary file 4 — Additional file 4. Manhattan plots of the body traits using imputation to higher density SNP dataset. [file 12864_2025_11865_MOESM4_ESM.zip › Suppl. 4. Figure A4 4 Manhattan plot for EL using imputed genome.jpeg]

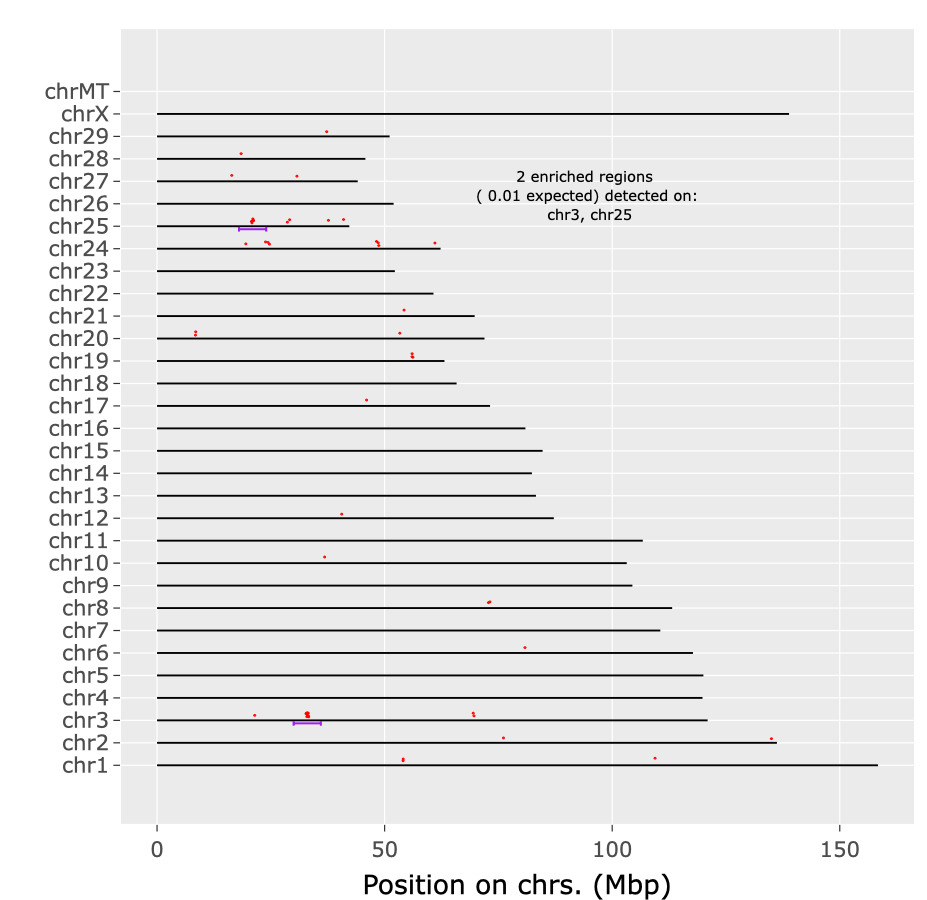

Supplement: Supplementary file 5 — Additional file 5. KEGG enrichment of candidate genes on BTA3 and BTA25. [file 12864_2025_11865_MOESM5_ESM.jpg]

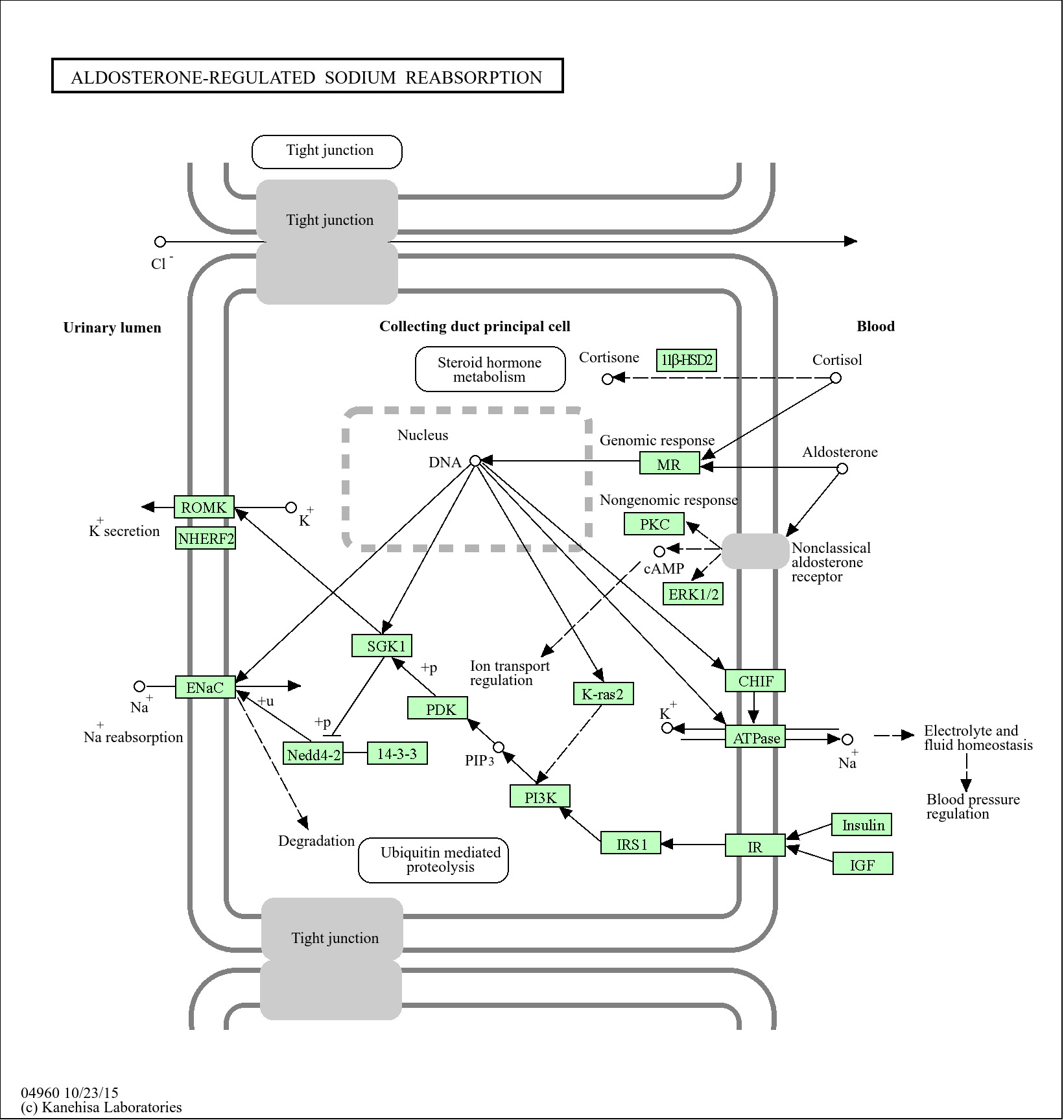

Supplement: Supplementary file 6 — Additional file 6. Aldosterone-regulated sodium reabsorption (a) and taste transduction (b) pathways. [file 12864_2025_11865_MOESM6_ESM.zip › Suppl. 6. Figure A6 1 aldosterone pathway.jpg]

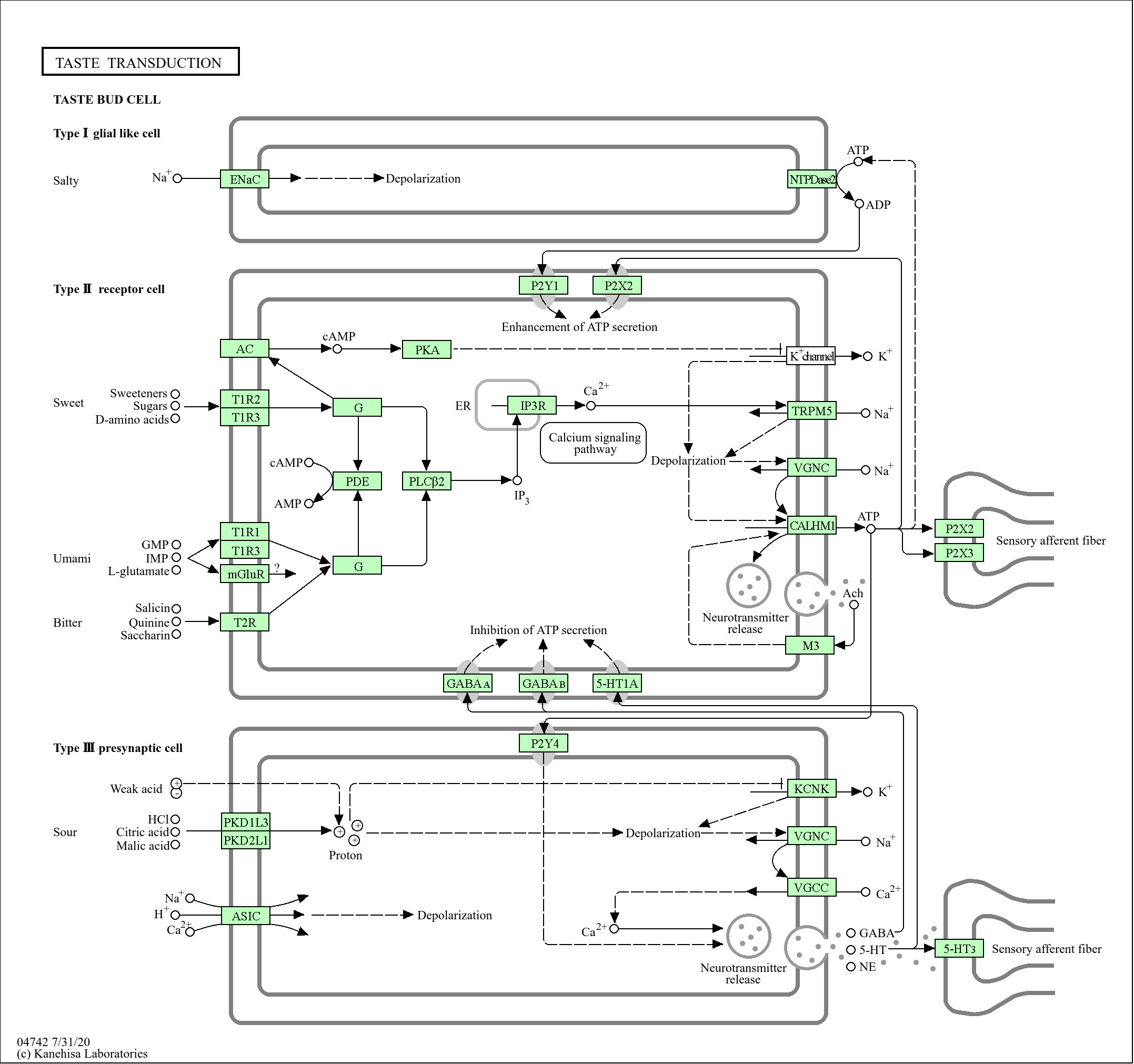

Supplement: Supplementary file 6 — Additional file 6. Aldosterone-regulated sodium reabsorption (a) and taste transduction (b) pathways. [file 12864_2025_11865_MOESM6_ESM.zip › Suppl. 6. Figure A6 2 taste pathway.jpg]
