# Supplementary material for: Genome-wide association analysis revealed novel candidate genes for body measurement traits in indigenous Gudali and crossbred Simgud in Cameroon
Source: BMC Genomics. 2025 Jul 14;26:664. doi: 10.1186/s12864-025-11865-7 (PMC12257737; doi:10.1186/s12864-025-11865-7)
Supplement: Supplementary file 7 — Additional file 7. Phenotypic distribution and ANOVA table. [file 12864_2025_11865_MOESM7_ESM.pdf]

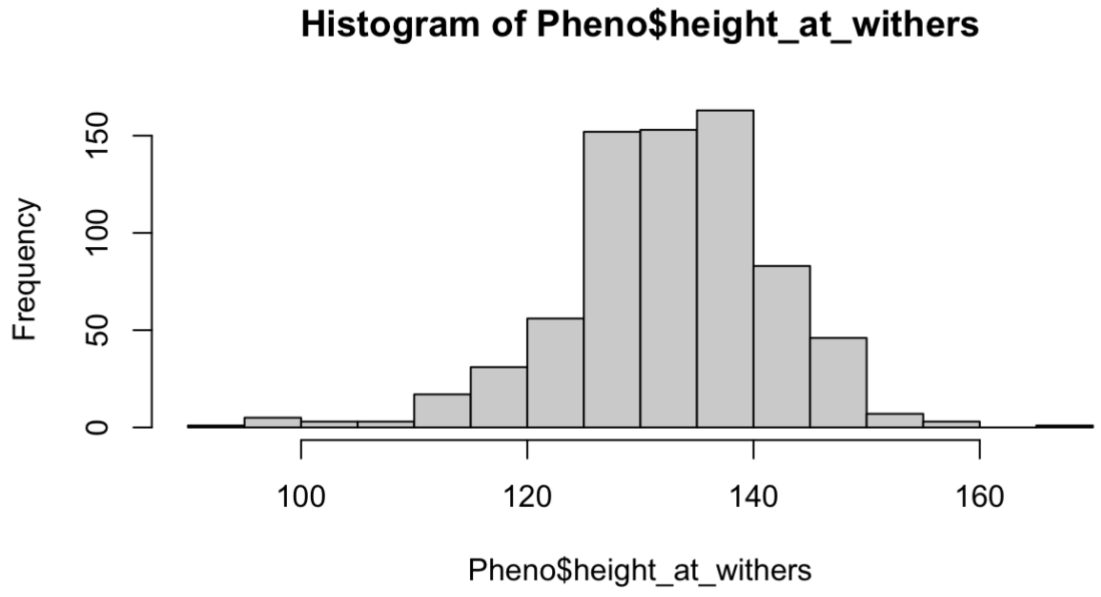

Histogram of Pheno$height_at_withers
Frequency
0
50
100
150
100
120
140
160
Pheno$height_at_withers

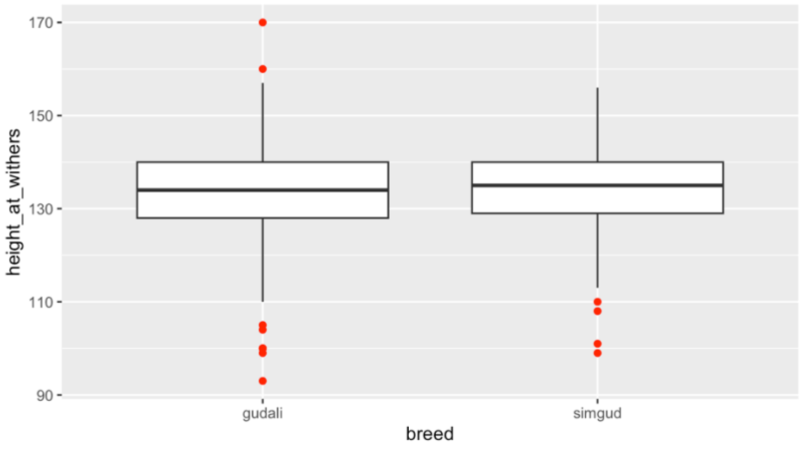

height_at_withers
170
150
130
110
90
gudali
simgud
breed

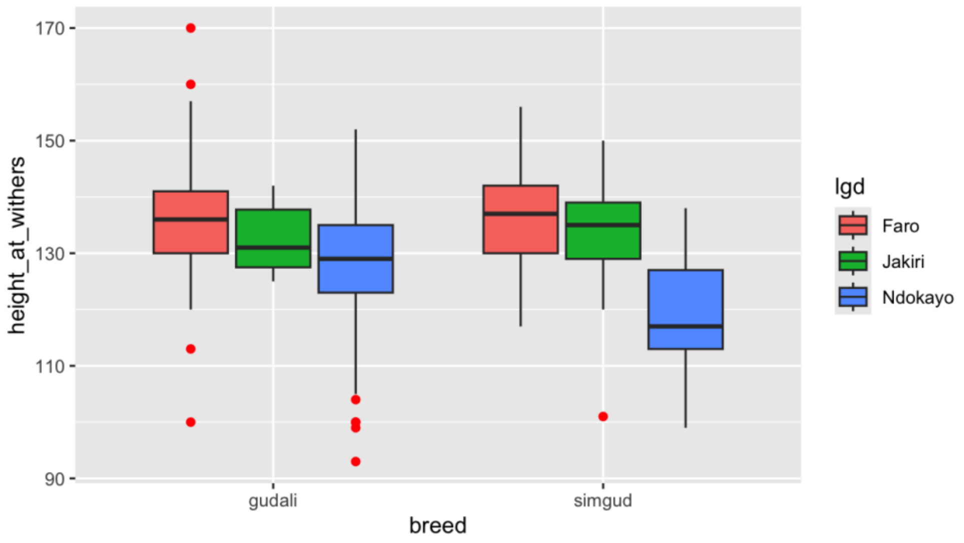

height_at_withers
170
150
130
110
90
gudali
simgud
breed
lgd
Faro
Jakiri
Ndokayo

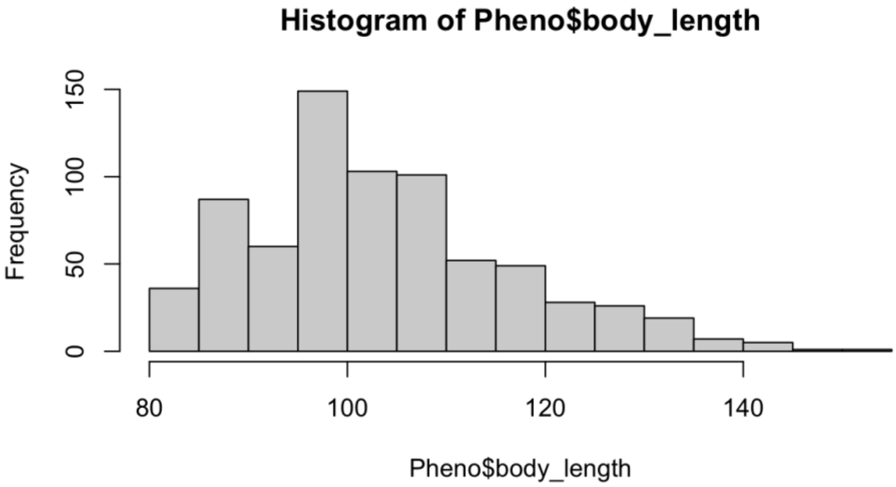
Histogram of Pheno$body_length
Frequency
0
50
100
150
80
100
120
140
Pheno$body_length

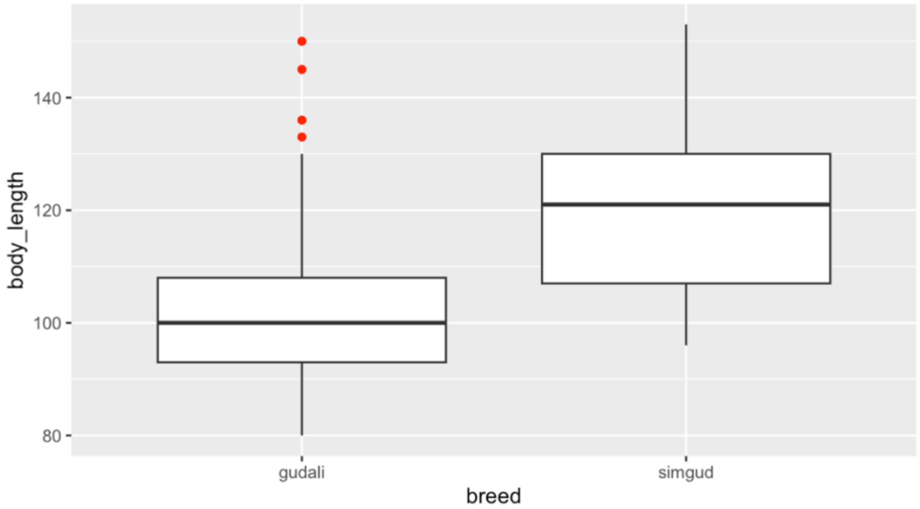
body_length
80
100
120
140
gudali
simgud
breed

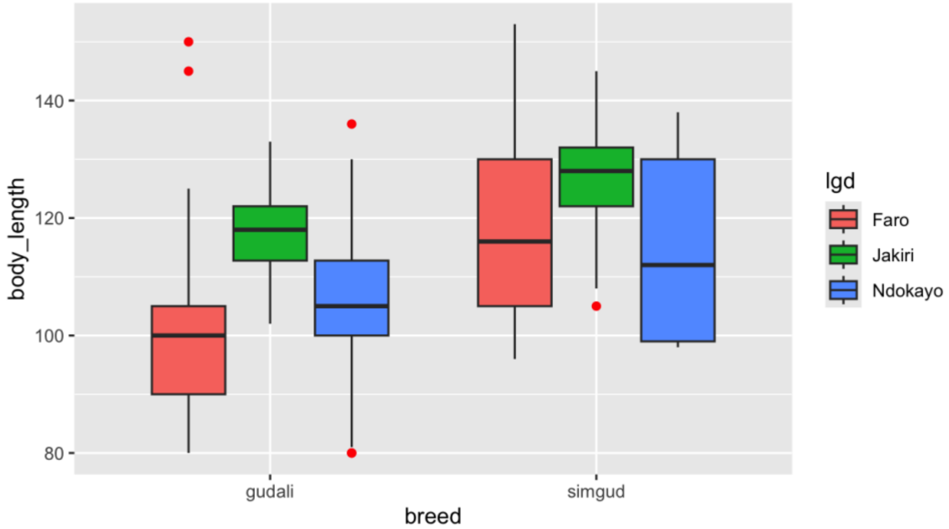
body_length
80
100
120
140
gudali
simgud
breed
lgd
Faro
Jakiri
Ndokayo

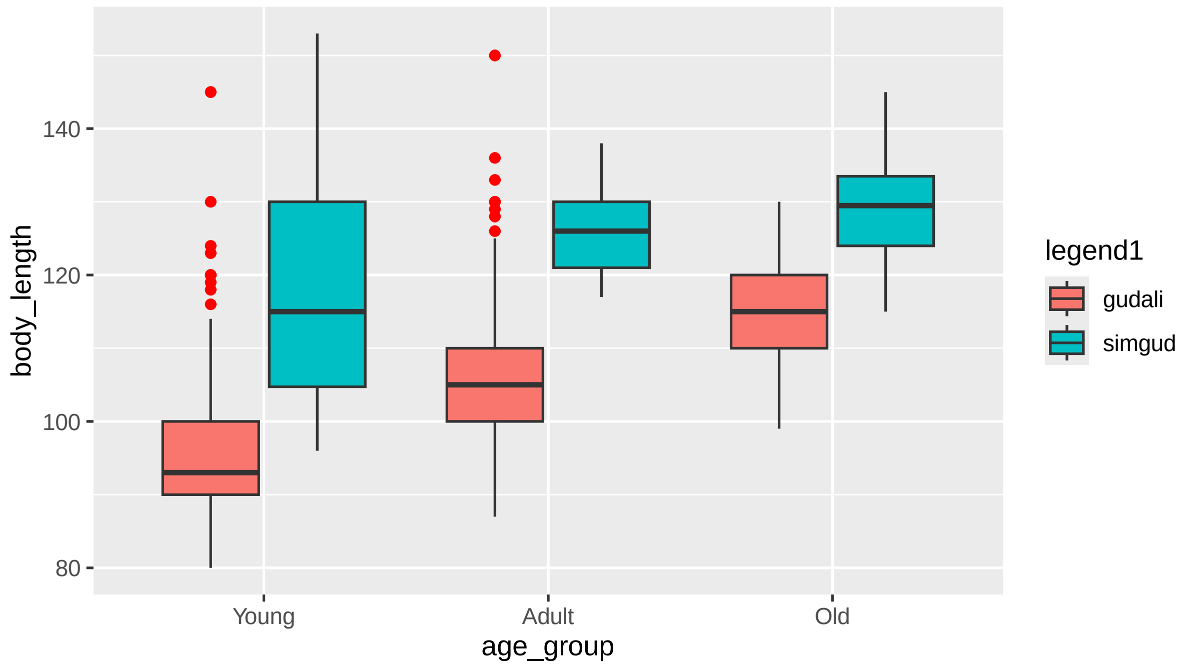
body_length
140
120
100
80
Young
Adult
Old
age_group
legend1
gudali
simgud

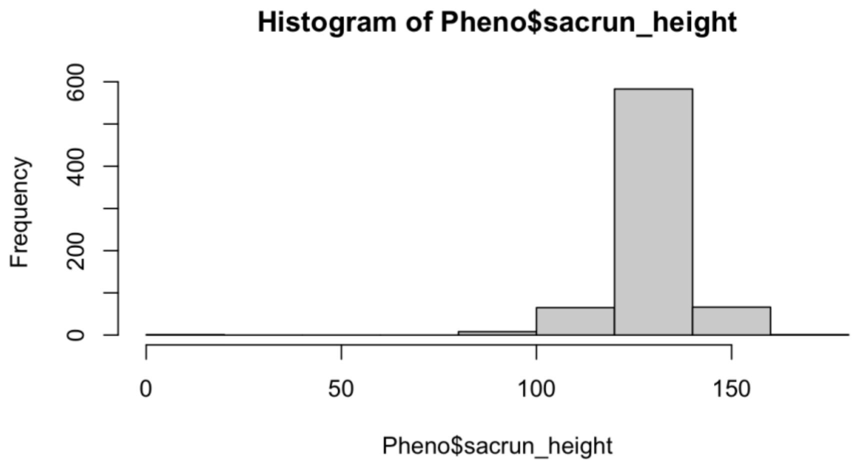
Histogram of Pheno$sacrun_height
Frequency
0
200
400
600
0
50
100
150
Pheno$sacrun_height

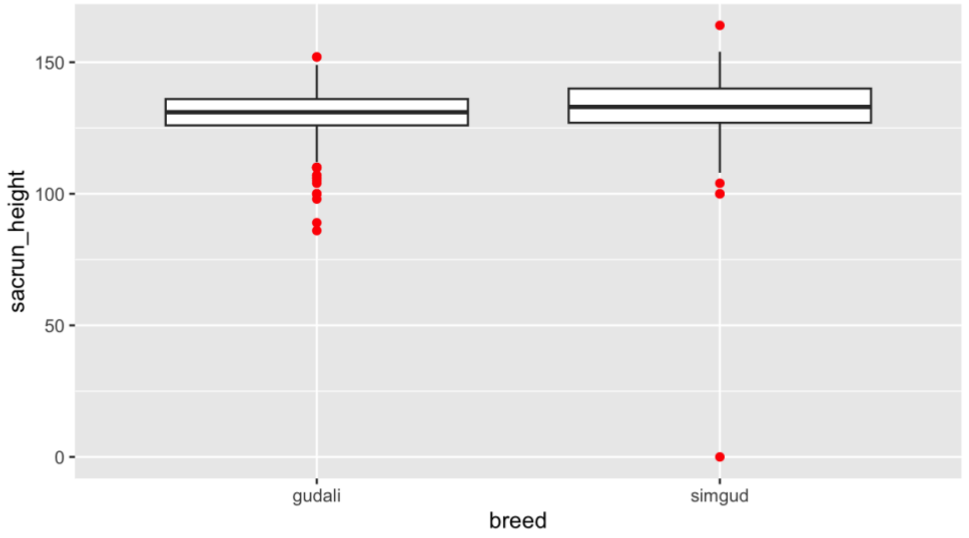
sacrun_height
150
100
50
0
gudali
simgud
breed

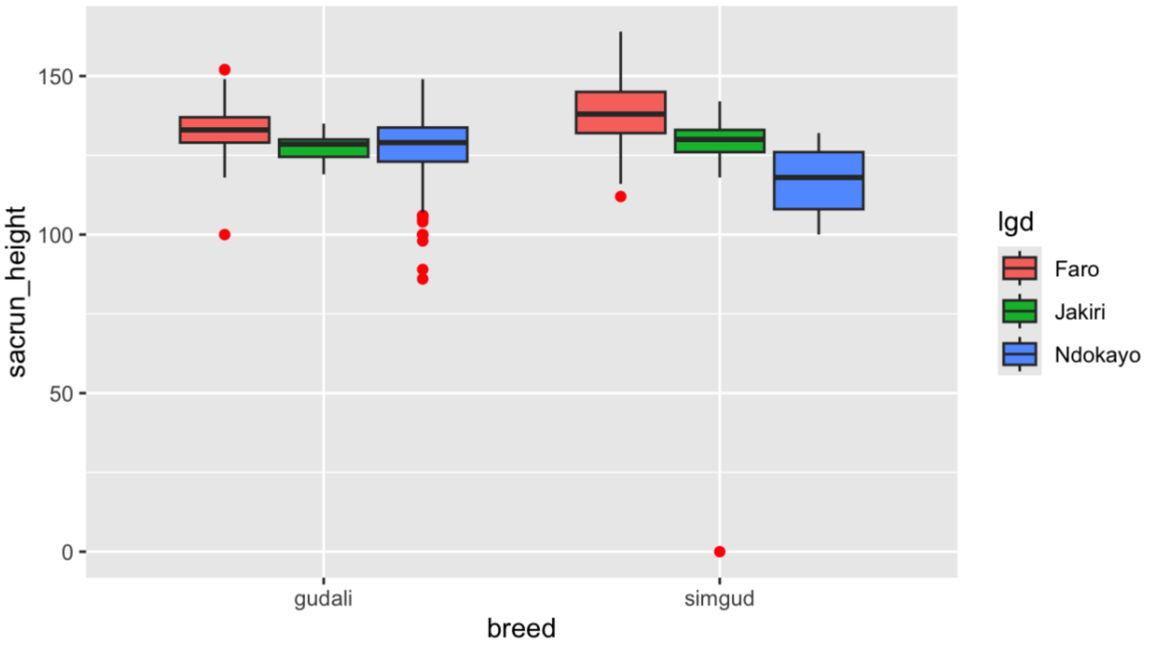
sacrun_height
150
100
50
0
gudali
simgud
breed
lgd
Faro
Jakiri
Ndokayo

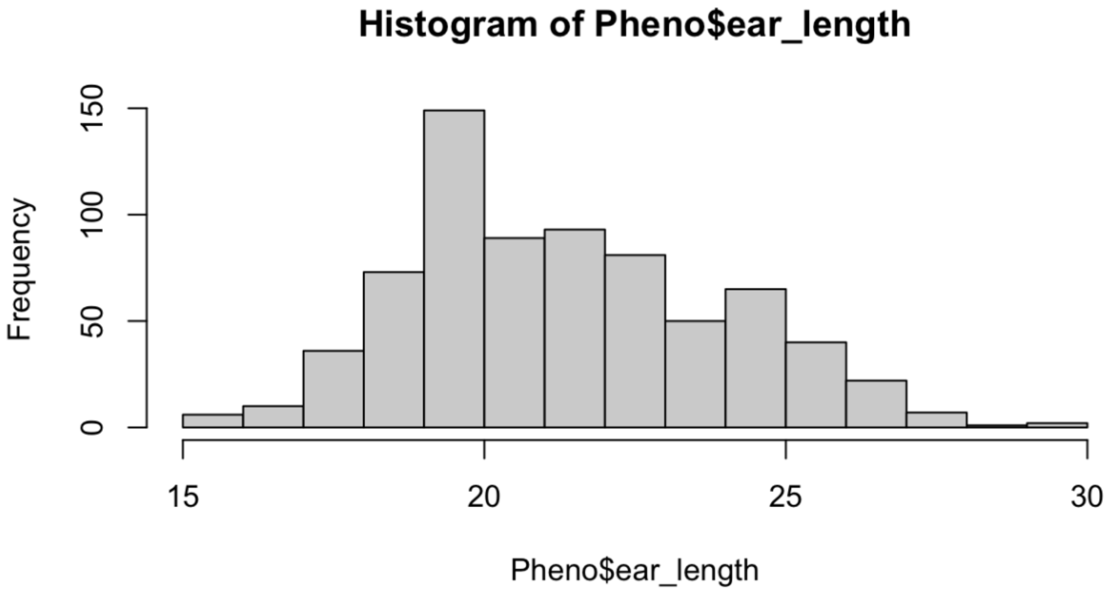
Histogram of Pheno$ear_length
Frequency
0
50
100
150
15
20
25
30
Pheno$ear_length

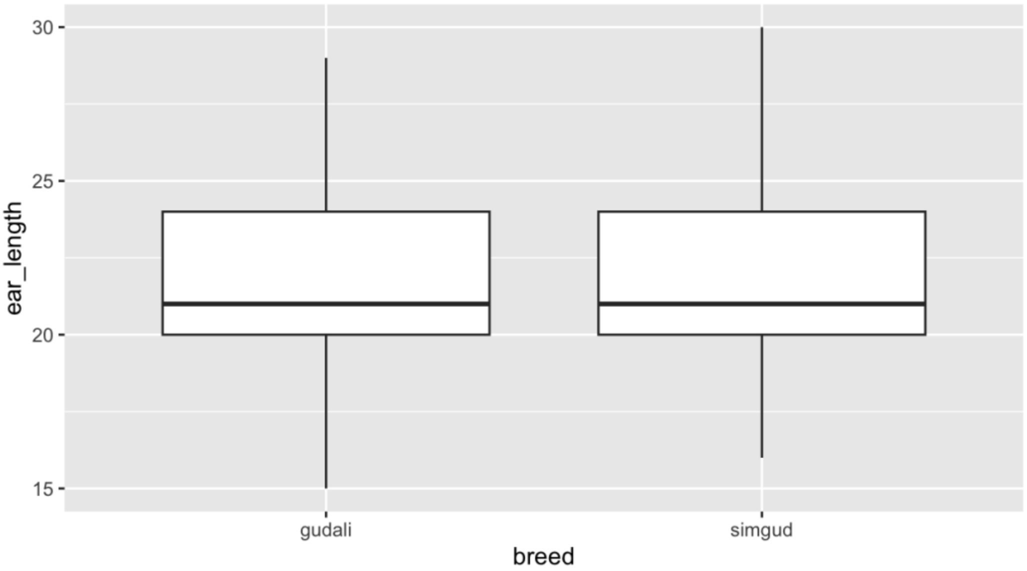
ear_length
15
20
25
30
gudali
simgud
breed

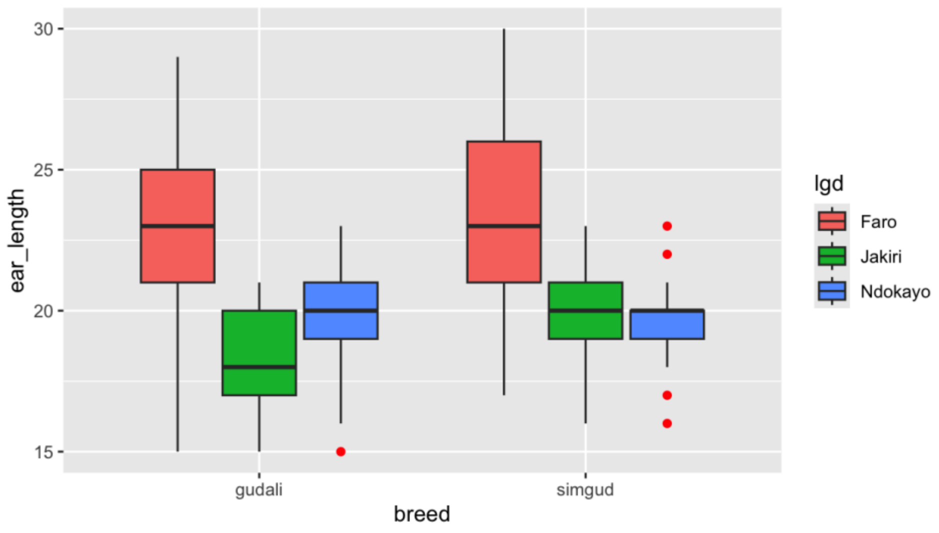

ANOVA: Effect of fix factors (breed, ranch, camp, herd, sex, age group) on body traits

Trait	Parameter	Df	Sum Sq	Mean Sq	F value	Pr(>F)
HAW	breed	1	49	49	0.83	0.362
	ranch	2	11128	5564	94.89	<2E-16***
	camp	6	5736	956	16.31	<2E-16***
	Herd	22	6332	288	4.91	3.2E-12***
	sex	1	326	326	5.55	0.019*
	age_group	2	52	26	0.45	0.641
	breed:ranch	2	60	30	0.51	0.601
	Residuals	687	40282	59		
Body length	breed	1	33366	33366	519.42	<2E-16***
	ranch	2	8997	4498	70.03	<2E-16***
	camp	6	32169	5361	83.46	<2E-16***
	Herd	22	6935	315	4.91	3.2E-12***
	sex	1	3	3	0.04	0.84
	age_group	2	242	121	1.88	0.15
	breed:ranch	2	99	50	0.77	0.46
	Residuals	687	44131	64		
Sacrum heigth	breed	1	198	198	2.48	0.11594
	ranch	2	8960	4480	55.94	<2E-16***
	camp	6	4059	676	8.45	7.3E-09***
	Herd	22	4413	201	2.5	0.00018***
	sex	1	772	772	9.64	0.00198**
	age_group	2	496	248	3.09	0.04597*
	breed:ranch	2	339	169	2.12	0.12142
	Residuals	687	55019	80		
Ear length	breed	1	5	5	1.25	0.263

Trait	Parameter	Df	Sum Sq	Mean Sq	F value	Pr(>F)
	ranch	2	1425	713	167.35	<2E-16***
	camp	6	48	8	1.87	0.084
	Herd	22	443	20	4.73	1.3E-11***
	sex	1	3	3	0.74	0.39
	age_group	2	21	10	2.44	0.088
	breed:ranch	2	14	7	1.68	0.187
	Residuals	687	2926	4		

ANOVA within breed (Simgud)

Trait	Parameter	Df	Sum Sq	Mean Sq	F value	Pr(>F)
Ear length	ranch	2	334.4	167.21	25.930	7.57e-10 ***
	camp	3	6.1	2.04	0.316	0.814
	herd	8	16.7	2.09	0.324	0.955
	Age group	2	0.6	0.28	0.043	0.958
	Residual	103	664.2	6.45		
Sacrum height	ranch	2	6185	3092.5	14.235	3.48e-06 ***
	camp	3	1082	360.7	1.660	0.1802
	herd	8	403	50.3	0.232	0.9842
	Age group	2	1937	968.3	4.457	0.0139 *
	Residual	103	22376	217.2		
Body length	ranch	2	2147	1073.6	9.431	0.000173 ***
	camp	3	6340	2113.3	18.565	1.06e-09 ***
	herd	8	1223	152.8	1.342	0.231147
	Age group	2	8	4.2	0.037	0.964108
	Residual	103	11725	113.8		
Heigth at wither	ranch	2	3335	1667.4	24.309	2.25e-09 ***
	camp	2	500	166.5	2.428	0.0697
	herd	8	961	120.2	1.752	0.0953
	Age group	2	231	115.5	1.684	0.1907
	Residual	103	7065	68.6		

ANOVA within breed (Gudali)

Trait	Parameter	Df	Sum Sq	Mean Sq	F value	Pr(>F)
Ear length	ranch	2	1115.6	557.8	144.961	< 2e-16 ***
	camp	6	43.1	7.2	1.865	0.0847
	herd	20	471.2	23.6	6.123	6.28e-15 ***

	Age group	2	19.6	9.8	2.551	0.0789
	Residual	574	2208.6	3.8		
Sacrum height	ranch	2	4927	2463.5	46.018	< 2e-16 ***
	camp	6	3088	514.7	9.615	4.30e-10 ***
	herd	20	3311	165.5	3.092	8.09e-06 ***
	Age group	2	20	9.9	0.184	0.832
	Residual	574	30728	53.5		
Body length	ranch	2	8257	4128	75.970	< 2e-16 ***
	camp	6	25398	4233	77.895	< 2e-16 ***
	herd	20	5957	298	5.481	5.82e-13 ***
	Age group	2	328	164	3.018	0.0497 *
	Residual	574	31192	54		
Heigth at wither	ranch	2	8708	4354	76.893	< 2e-16 ***
	camp	6	4945	824	14.554	1.70e-15 ***
	herd	20	5670	283	5.007	1.65e-11 ***
	Age group	2	0	0	0.004	0.996
	Residual	574	32502	57		
